# Metabolic memory in diabetic complications: evidence for persistent cellular states and limits of therapeutic reversal

**DOI:** 10.3389/fendo.2026.1954392

**Published:** 2026-10-05

**Authors:** Yulan Cai, Shuangyu He, Feng Zeng, Chuyu Liu

**Affiliations:** 1 Department of Endocrinology, The Second Affiliated Hospital of Zunyi Medical University, Zunyi, Guizhou, China; 2 Department of Endocrinology and Metabolism, Affiliated Hospital of Zunyi Medical University, Zunyi, Guizhou, China; 3 Department of General Practice, The Second Affiliated Hospital of Zunyi Medical University, Zunyi, Guizhou, China; 4 Thyroid and Breast Surgery, The Second Affiliated Hospital of Zunyi Medical University, Zunyi, Guizhou, China

**Keywords:** cellular senescence, diabetic complications, epigenetics, immunometabolism, metabolic memory, mitochondrial dysfunction, tissue repair, trained immunity

## Abstract

Metabolic memory is defined here as an exposure-history-dependent cellular phenotype that persists beyond immediate carry-over after verified withdrawal of the initiating exposure, principally high glucose and is accompanied by a specified functional impairment or an altered response to the same defined secondary stimulus. This operational definition separates cellular memory from clinical legacy effects, continuing local stimulation and residual structural injury. We critically examine evidence from longitudinal clinical studies, human biospecimens, animal models and controlled cell experiments, distinguishing molecular persistence, measured signaling activity, causal maintenance and treatment-free durability. DCCT/EDIC and UKPDS establish lasting benefits of earlier glycemic management but do not independently identify cellular memory as the mechanism. Experimental evidence supports selected endothelial redox circuits, epigenetic regulation and hematopoietic progenitor training; human causal evidence is substantially more limited. Persistent chromatin marks do not necessarily imply continuously active signaling, and latent priming may become apparent only after rechallenge. In kidney, cardiovascular, retinal and wound disease, candidate cellular memory coexists with hypoxic or inflammatory niches and structural loss. Pancreatic β-cell plasticity is considered a comparative endocrine model rather than an established complication-memory circuit. The proposed interactions among epigenetic, mitochondrial, metabolic and inflammatory mechanisms remain a framework supported by selected local feedback loops, not a universally demonstrated network. Therapeutic evidence is organized into prevention, modification or suppression of established abnormalities, and sustained molecular and functional recovery after treatment cessation after intervention withdrawal. The latter remains unestablished in patients for the mechanisms reviewed. Early glycemic and multifactorial management remains essential, while mechanistic translation requires explicit recovery, perturbation and post-treatment follow-up designs.

## 1 Introduction

The relationship between glycemic exposure and diabetic complications is not adequately captured by a single contemporaneous glycated hemoglobin value. In the Diabetes Control and Complications Trial (DCCT), intensive treatment reduced retinopathy, nephropathy and neuropathy in type 1 diabetes; during Epidemiology of Diabetes Interventions and Complications (EDIC) follow-up, these benefits persisted after the original separation in glycated hemoglobin had substantially narrowed. Cardiovascular benefit also became evident over prolonged observation (1–4). A comparable legacy effect was observed after the UK Prospective Diabetes Study, and the extended UKPDS 91 analysis showed that benefits associated with early glycemic and metformin allocation remained detectable for up to 24 years (5, 6). Observational cohorts likewise indicate that early glycemic exposure and visit-to-visit variability predict later outcomes beyond a single current measurement (7, 8).

These findings do not diminish the importance of present-day metabolic control. Our principal focus is previous hyperglycemia. Here, cellular metabolic memory means an exposure-history-dependent phenotype that persists beyond immediate molecular carry-over after verified withdrawal of the initiating metabolic stress and is coupled to dysfunction or an altered response to a standardized challenge. Evidence that an active mechanism maintains this phenotype requires perturbation during recovery. The clinical legacy effect denotes a lasting difference in outcomes after earlier treatment; hyperglycemic memory specifies high glucose as the initiating exposure. Broader lipid- or inflammation-induced states are included only when exposure withdrawal and functional persistence are tested. Hypoxia and inflammation that continue after glucose lowering are ongoing stimuli, not automatically additional forms of memory.

DCCT/EDIC and UKPDS establish clinical legacy effects, not cellular mechanisms (1–6). Earlier treatment may prevent irreversible lesions, preserve vascular reserve or reduce the cumulative burden of injury. A continuing separation in cumulative event curves can also reflect events prevented earlier and does not require a permanently elevated contemporary hazard. Narrowing between-group HbA1c does not mean that all participants achieved normal glucose or that local stress disappeared. The trials are compatible with cellular memory, but do not distinguish it from these alternative explanations.

Human observations also identify specific biological correlates of previous glycaemia. A DCCT/EDIC analysis of blood DNA from 499 participants related methylation to preceding HbA1c and subsequent complications over 18 years (134). Its statistical mediation analysis supports a potential contribution of methylation but does not establish that editing a CpG site would improve patient outcomes. In another DCCT study, skin collagen glycation and carboxymethyllysine in 211 participants predicted later retinopathy and nephropathy progression (135). This provides a concrete alternative or complementary explanation involving accumulated tissue protein modification, rather than proving that all clinical legacy effects arise from an intracellular memory circuit.

Mechanistic evidence must be distinguished by experimental setting. Endothelial glucose-switch experiments demonstrate retained RELA transcription and NF-κB target expression (9), whereas DCCT/EDIC blood studies associate historical glycaemia with histone acetylation and DNA methylation, including TXNIP, without proving causal maintenance in patients (10, 11). Retinal mitochondrial injury persists in experimental recovery models (12). Patient-derived diabetic foot ulcer fibroblasts retain methylation abnormalities during culture (13). For HSPCs, controlled high-glucose withdrawal and transmission through differentiation were demonstrated mainly in umbilical-cord-blood-derived CD34-positive cells; diabetic patient bone-marrow samples provided a separate clinical comparison (14). These observations support different inferences and should not be merged into a single claim of demonstrated human cellular causality.

We examine metabolic memory as a set of potentially modifiable, lineage-dependent states. This is a working framework, not a demonstrated universal self-sustaining network. Some cells retain active stress outputs, whereas others retain a primed response with little basal activation. Mitochondrial injury, autocrine signaling and an abnormal extracellular matrix can also prolong dysfunction; experiments must distinguish acquired cellular persistence from continued environmental instruction. **Figure 1** illustrates these alternative trajectories and the boundaries of the proposed framework.

**Figure 1:**
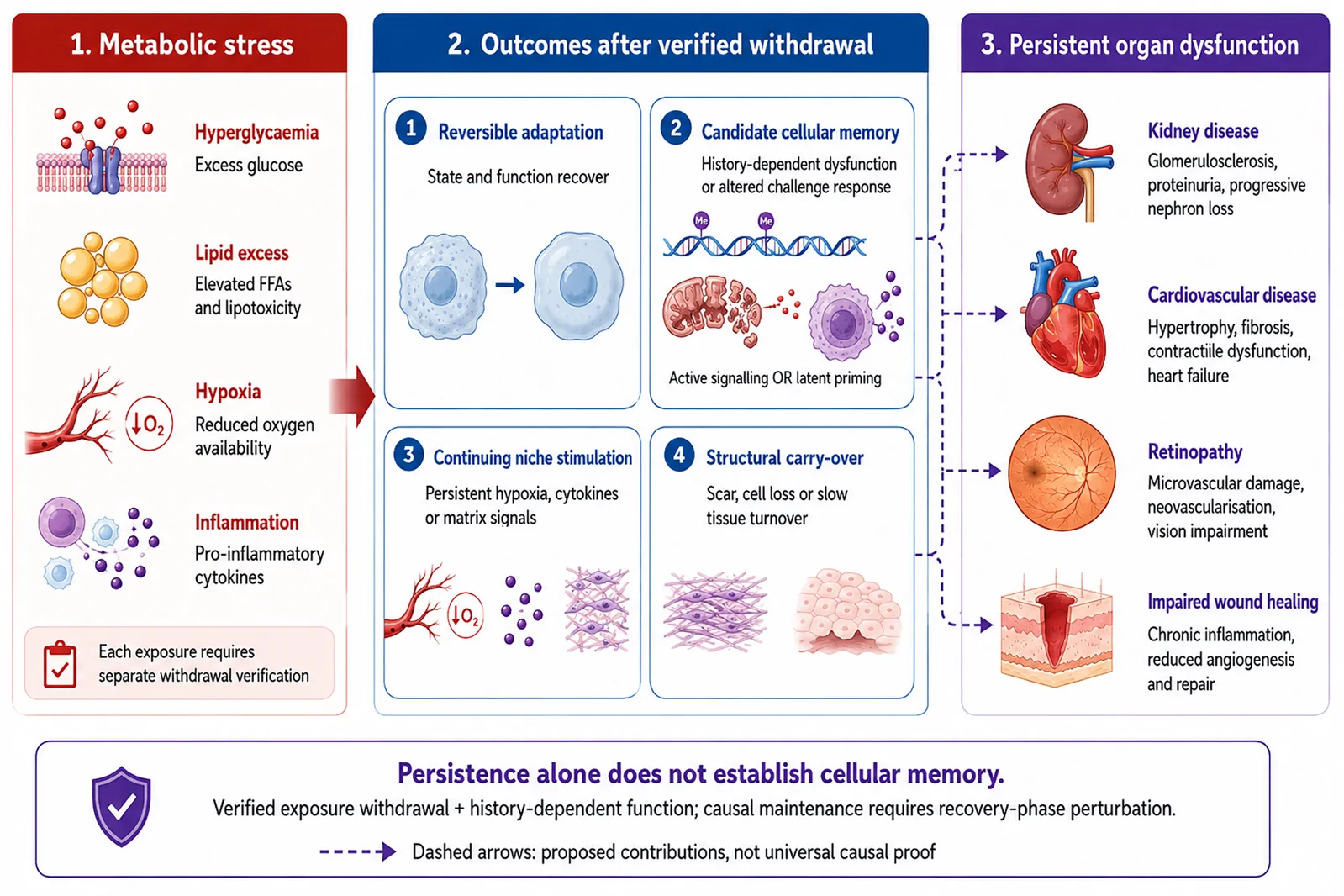
Conceptual trajectories after metabolic stress. Candidate cellular memory is distinguished from reversible adaptation, continuing niche stimulation and structural injury. Persistence is considered a functional history effect only after the initiating exposure has been withdrawn and immediate carry-over addressed. Active signaling and latent priming are separate possible phenotypes. Arrows indicate proposed relationships and do not imply that all transitions or a common network have been demonstrated in patients.

This is a critical narrative review rather than a systematic review or meta-analysis. Theoriginal account reported PubMed and Google Scholar searches through July 2026, butdatabase-specific strings, screening counts and selection logs are not available for reconstruction.We therefore do not claim that the original selection process was reproducible or independentlyscreened. The preceding version reported a documented, focused PubMed search performed on 8September 2026, followed by targeted assessment of representative primary studies and sourcechecking of the original references. The retained historical portion of **Appendix A** provides the reportedexact query, publication-date limits, selection criteria, retrieval count, study-selection rationaleand limitations. Studies of ongoing diabetes or cross-sectional disease were retained for contextbut were not treated as direct evidence of post-withdrawal memory. Results showing recovery,incomplete persistence or failure of an intervention were used to qualify the framework rather thanexcluded for their direction. For the present update, targeted searches of PubMed-indexed recordsand checks against accessible primary sources were undertaken on 14 September 2026. The additionalsource checks and Google Scholar access limitation are described in **Appendix A7**. The earlier retrieval counts are not counts from this update.

## 2 Defining metabolic memory as a testable biological state

### 2.1 Memory, adaptation, and structural damage are not equivalent

Persistence is necessary for metabolic memory, but persistence alone is not sufficient. An abnormality may remain because the initiating stress has not been fully removed, because a protein or extracellular structure turns over slowly, because cells have been lost, or because an active circuit continues to maintain a pathological state. These possibilities have different biological and therapeutic implications. Without explicit boundaries, metabolic memory risks becoming a loose label for any diabetic abnormality that fails to normalize immediately.

Four conditions should be separated. An acute response resolves after stress removal. A reversible adaptation supports survival during stress. A candidate memory phenotype remains after a defined recovery period and is coupled to dysfunction or altered challenge responsiveness. Structural injury, including capillary loss, glomerulosclerosis, myocardial scar or denervation, can persist without an active memory programme. Long-lived glycated collagen exemplifies slowly turning-over extracellular material (15). Persistent stress signaling is not an alternative diagnosis to memory: it may be its maintenance mechanism, but only if prior exposure, verified withdrawal, ongoing activity and a history-dependent functional effect are demonstrated. Otherwise, continued stress or damage remains sufficient to explain the observation.

A stringent experiment defines exposure intensity and duration, confirms withdrawal and measures recovery at multiple time points appropriate to cell division and molecular turnover. It couples molecular measurements to function, includes never-exposed and continuously exposed controls, and perturbs candidate mechanisms after persistence has formed. Residual glucose, lipids, cytokines, osmotic effects, cell selection and tissue loss require explicit consideration. A quiescent but primed cell can satisfy the functional criterion through an abnormal standardized secondary response; it should not be described as constitutively activated. **Box 1** separates evidence for persistence from the additional requirements for causal maintenance and sustained recovery after the experimental treatment ends.

Box 1 **Operational criteria and evidence categories for metabolic memory**

**Table:** 

Operational definition: a history-dependent cellular phenotype persists beyond immediate carry-over after verified withdrawal of the initiating metabolic stress and is coupled to dysfunction or altered response to the same defined secondary stimulus.
Evidence for a candidate memory phenotype
1. Define exposure intensity, duration and identity. Glucose, lipids, oxygen and inflammatory ligands are separate variables.
2. Verify withdrawal locally as well as systemically. Improved HbA1c alone does not establish normoglycaemia or removal of tissue stress.
3. Include never-exposed and continuously exposed controls, repeated recovery time points and an interval appropriate to turnover and cell division.
4. Couple a retained molecular state to function or challenge response. Separate directly measured signalling activity from expression or chromatin accessibility.
5. Evaluate alternatives: residual ligand, selection or altered cell composition, slow turnover, cell loss, scar and continuing local inflammatory, hypoxic or matrix-derived stimulation.
Additional evidence for causal maintenance
6. Perturb after persistence has formed. Test target engagement and downstream function. Preventing induction establishes encoding dependence, not maintenance dependence. Rescue or orthogonal perturbation strengthens specificity.
Additional evidence for sustained molecular and functional recovery after treatment cessation
7. Confirm molecular and functional recovery after intervention clearance, with follow-up appropriate to the model. Standardized rechallenge strengthens evidence that response bias has normalized.
Non-ranked evidence categories
Longitudinal clinical studies; human biospecimens or patient-derived cells; animal experiments; controlled cell experiments. Human cell origin does not change an in vitro experiment into clinical causal evidence.
Dimensions of inference
Association; molecular or functional persistence; measured signalling activity; perturbation of encoding or maintenance; treatment-free durability. These are distinct descriptors, not ordinal levels or a validated quality score.

The terms used here refer to specific observations or experimental procedures, not separate disease entities. Persistent dysfunction means a named impairment that remains at the stated recovery time, such as reduced endothelium-dependent relaxation; a methylation difference alone is a molecular finding. A standardized challenge applies the same secondary stimulus, dose, duration and assay conditions to previously exposed and control cells, for example measuring cytokine release after inflammatory stimulation. Residual structural injury means persisting tissue damage such as glomerulosclerosis, capillary loss, scar or denervation. Causal maintenance means that perturbing a regulator after the abnormality has developed changes a relevant downstream outcome; preventing injury before high glucose does not establish this. Intervention withdrawal means ending the experimental treatment and allowing its pharmacological or target effects to subside. It is distinct from glucose removal and does not require withdrawal of indicated diabetes care. Sustained recovery after treatment cessation requires both specified molecular and functional improvement to remain during subsequent observation. It does not imply complete organ repair or erasure of exposure history.

### 2.2 Evidence categories and dimensions of inference

We distinguish four non-ranked evidence categories: longitudinal clinical studies; human biospecimens and patient-derived cells; animal experiments; and controlled cell experiments. Human origin and experimental design are recorded separately: a human endothelial cell line or cord-blood glucose-switch experiment remains *in vitro* evidence, not proof in treated patients. Within each category we distinguish association, molecular or functional persistence, directly measured signaling activity, causal perturbation and treatment-free durability. Causal evidence also depends on timing: prevention of encoding is different from interference with established maintenance. These dimensions are not a numerical score, and no formal risk-of-bias assessment was undertaken. **Table 1** applies them to representative mechanisms; **Table 2** distinguishes direct post-normalization evidence from indirect cellular observations.

**Table 1:** Landmark clinical and experimental evidence for metabolic memory.

Study and origin	Withdrawal or recovery	Persistent finding and activity	Causal inference	Durability and main limit
DCCT/EDIC and UKPDS (1–6) Longitudinal clinical	Treatment-group glycemic differences narrowed; participants were not uniformly normoglycemic.	Long-term differences in clinical events; no direct cellular signaling assay.	Randomized early-treatment effect with observational follow-up; cellular mechanism unresolved.	Clinical legacy, not demonstrated cellular reset. Prevented structural injury and cumulative exposure remain explanations.
DCCT/EDIC epigenomics (10, 11) Human biospecimens	Historical treatment separation attenuated.	Histone acetylation and DNA methylation associated with prior exposure; no continuous pathway activity established.	Association and selected longitudinal molecular persistence; no locus-specific intervention in patients.	Persistence of a mark does not establish causal maintenance or recovery sustained after experimental treatment cessation.
SETD7/RELA (9) Endothelial culture and mouse experiment	16 h high glucose, then up to 6 d normal-glucose culture. The accompanying transient-hyperglycemia mouse experiment used otherwise non-diabetic animals.	H3K4me1, RELA transcription and NF-κB target expression remain elevated.	Experimental SETD7/redox dependence; distinguish encoding prevention from delayed maintenance intervention.	No sustained functional recovery after the intervention ends established.
PKCβII/p66Shc/ROS (34) Cells and mice	Normal glucose or insulin-mediated glucose correction.	Persistent activation and mitochondrial translocation, ROS and reduced NO function.	Silencing at normalization improves vascular function; a defined feedback module is supported.	Intervention-associated rescue; treatment-free durability not established.
Retinal DNMT/mtDNA/MMP9 (16) Human retinal endothelial cultures and rats	Cells: 20 mmol/L glucose for 4 d, then 5 mmol/L for 4 or 8 d; rats: 3 months poor then 3 months good control.	DNMT activity, methylation-related changes and transcription persist initially; selected endpoints improve with longer recovery.	Recovery-phase DNMT inhibition modifies retained abnormalities.	Broad drug action limits locus specificity; natural recovery qualifies claims of permanence.
DNMT1/IMPDH2 (17) Cells, diet-switched mice and human samples	Glucose-switched cultures; dietary intervention in mice.	Sustained nucleotide-related changes, oxidative/inflammatory outputs and excess sprouting. Human measurements are associative.	DNMT and IMPDH2 inhibition supports experimental coupling.	Diet switch is not proof of complete niche normalization; no clinical causal or withdrawal reset evidence.
CD34-positive HSPCs (14) Human cell experiment plus patient comparison	Cord-blood cells: 20 d high glucose, 3 or 10 d recovery; myeloid differentiation.	Persistent NF-κB transcriptional activity, chromatin changes and progeny response bias.	Controlled *in vitro* persistence. Patient bone-marrow signatures corroborate association.	Patient cells were not a longitudinal normalization cohort; clinical organ-level causality unproven.
Diabetic marrow transfer (20) Mice and cultured macrophages	Differentiation at physiological glucose; transfer to normoglycemic Ldlr-deficient recipients.	Primed chromatin and inflammatory challenge responses; increased recipient atherosclerosis.	Transfer supports a hematopoietic contribution; RUNX1 inhibition tested *in vitro*.	Whole marrow and recipient atherogenic conditions limit HSPC specificity; dominance unproven.
HSC/DNMT1 and healing (53) Mouse HSC experiments	Transplantation and wound challenge outside the original diabetic donor.	Abnormal myeloid differentiation and impaired repair.	Transfer and DNMT1 manipulation support a causal contribution in this model.	Not proof of dominant human HSPC memory or sustained recovery after experimental treatment cessation.
DFU fibroblasts (13, 56) Patient-derived cultures	Passaging and reconstructed skin outside original wound.	Methylation differences and poor tissue-support function. Continuous signaling not directly established by methylation.	Persistence outside the wound; exposure attribution and locus causality incomplete.	Culture selection and donor factors remain; same-lineage recovery sustained after experimental treatment cessation not established.
Tubular DNMT1/p21 (25) Cells, mice and human samples	Glucose switch; insulin or SGLT2 treatment in mice; improved glycaemia in human observations.	Sustained p21/senescence and renal dysfunction; human p21 persistence or association.	Experimental DNMT1/p21 manipulation and aPC-related interventions modify disease.	Causality is preclinical; ongoing renal crosstalk and post-treatment durability unresolved.
Macrophage p66Shc (24) Cells and mice	Glucose normalization; recovery-phase aPC in culture.	p66Shc, ROS and proatherogenic phenotype; promoter hypomethylation.	p66Shc inhibition and DNMT1-dependent aPC effects support maintenance.	Glucose versus lipid specificity reported; intervention clearance and sustained recovery after experimental treatment cessation not established.
CaMK2a/O-GlcNAc and vesicles (43) Cells and mice; human association	Glucose correction; vesicle transfer to healthy recipients.	Continued CaMK2a activity and vesicle release; transferred cardiac dysfunction.	Specific experimental feedback and transfer; patient vesicles correlate with dysfunction.	Recipient exposure to vesicles continues to be an input; no universal or human causal network proven.
NRF2 activation (27) Human endothelial cultures	30 mmol/L glucose for 4 d, followed by 5.5 mmol/L for 4 d.	Persistent RNA and chromatin accessibility; sulforaphane modifies persistent RNA-expression and chromatin-accessibility differences.	Intervention supports molecular state modification; enhancer-specific causality not isolated.	Transcript/accessibility recovery is not equivalent to durable signaling and functional reset.
DNMT1/Ang-1/NF-κB (26) Cells and mice	A defined period of high-glucose exposure followed by glucose correction in experimental cells or mice; the exact protocol is study-specific (26).	Ang-1 methylation/repression, sustained NF-κB-related dysfunction and impaired healing.	DNMT1 intervention connects epigenetic regulation to vascular outcomes.	Preclinical mechanistic support; treatment-free durability not established.
Recovery counterexamples (16, 47, 92) Cell or animal models	Longer glucose recovery; delayed insulin; weight loss after diet switch.	Selected retinal methylation changes recover; inflammation can normalize despite synaptic abnormalities; most hepatic transcription/enhancer changes reverse.	Shows endpoint, tissue and time dependence.	Residual disease is not evidence that every pathway remains active.
Aortic single-cell profiling (87) Diabetic mice	6 weeks of dapagliflozin.	Persistent SMC transcription/accessibility with partial improvement of other programmes.	Molecular persistence; motif or pathway enrichment is not direct biochemical activity.	Continued obesity or niche changes may contribute; no specific maintenance intervention or recovery sustained after experimental treatment cessation.

Study origin and inference are assessed separately. Clinical legacy, molecular persistence, direct signaling activity, perturbation of maintenance and treatment-free durability are distinct findings. A human cell experiment is labelled *in vitro*; patient association is not a causal intervention. The table is a representative critical comparison, not a ranked quality scale or exhaustive evidence census.

All original study entries are retained to preserve the scope of the review. The glucose-related experiments should be read separately from the explicitly labelled comparative diet-reversal evidence in the recovery row; that comparison does not establish a mechanism after glycemic correction in diabetes. Persistent chromatin or transcript measurements indicate molecular persistence, whereas functional rescue after a delayed intervention offers additional mechanistic support. None is automatically evidence of sustained recovery after experimental treatment cessation.

DCCT, Diabetes Control and Complications Trial; DFU, diabetic foot ulcer; EDIC, Epidemiology of Diabetes Interventions and Complications; HSPC, hematopoietic stem and progenitor cell; mtDNA, mitochondrial DNA; STZ, streptozotocin; UKPDS, UK Prospective Diabetes Study.

**Table 2:** Cell-type-specific reprogramming in diabetic complications.

Cell type and normal role	Disease-associated phenotype and candidate regulators	Post-normalization evidence	Activity or functional evidence	Reversibility and limits
Endothelium Barrier, NO and adaptive angiogenesis	Senescence, EndMT, redox dysfunction or model-dependent excessive sprouting; SETD7/RELA, SIRT1/p300, p66Shc, DNMT1 (9, 17, 19, 21, 34, 52).	Direct in glucose-switch cells and selected animal models; patient causality limited.	NO/vasorelaxation, senescence, inflammatory outputs and angiogenesis measured in specific models.	Recovery-phase rescue supports modification. Treatment-free sustained recovery after experimental treatment cessation not established (16, 17, 19, 34).
HSPCs Self-renewal and balanced lineage output	Myeloid bias or altered differentiation; SETD7, RUNX1-associated accessibility and DNMT1 (14, 20, 53).	Direct in controlled cord-blood cultures and mouse transfer; patient evidence mainly associative.	NF-κB transcriptional activity in cell model, progeny responses and transferred organ effects.	Transferability does not prove dominance. Human HSPC reset and post-withdrawal durability unproven.
Monocytes and macrophages Host defense and resolution	Exaggerated secondary responses or impaired resolution; glycolytic/mevalonate pathways, NF-κB and p66Shc (20, 24, 39–41, 54).	Direct in defined glucose-training or recovery experiments; other studies concern active exposure.	Cytokine challenge responses and proatherogenic functions; latent priming need not mean basal activation.	aPC modifies experimental p66Shc state; established human diabetic training and sustained recovery after experimental treatment cessation insufficiently tested.
Fibroblasts Matrix turnover and repair	Poor wound support or context-specific fibrosis; methylation, TGF-β/mechanosensing and TNF-α/SETDB2 (13, 56, 59, 60).	Direct ex vivo persistence in DFU fibroblasts; mechanosensing/active-disease results are indirect for memory.	Defective repair support in reconstructed tissue. Methylation alone is not an activity assay.	Pluripotency/vascular conversion demonstrates plasticity (57, 58), not necessarily reset of the original mature lineage.
β cells Glucose-responsive insulin secretion	Identity loss, adaptation or senescence; PDX1, MAFA, NKX6.1, FOXO1 and mitochondrial/ER stress (61–68, 71).	Comparative endocrine model; human post-normalization circuitry insufficiently established.	Experimental identity and secretion recovery; human identity studies are mainly cross-sectional.	Redifferentiation after metabolic relief occurs in models (69, 70). Same circuitry as complication memory is not demonstrated.
Keratinocytes Migration and barrier renewal	Impaired migration/differentiation; FOXO1 target changes, autophagy, ROS and inflammatory crosstalk (72–82).	Direct lineage-specific post-normalization memory not established by the cited wound studies.	Active-wound or high-glucose functional defects; omics states do not prove persistence after withdrawal.	Modification during active diabetes is reported. Durable reset and independence from the wound niche remain unproven.

Direct post-normalization evidence, indirect disease association and unestablished persistence are explicitly distinguished. Intervention-associated improvement does not establish recovery sustained after experimental treatment cessation. Phenotypes are lineage- and context-dependent; β cells are included only as a comparative endocrine model.

AGEs, advanced glycation end-products; ECM, extracellular matrix; EndMT, endothelial-to-mesenchymal transition; HSPC, hematopoietic stem and progenitor cell; ROS, reactive oxygen species; SASP, senescence-associated secretory phenotype.

## 3 Encoding and stabilization of cellular memory

Epigenetic, mitochondrial, metabolic and inflammatory processes may interact during memory formation and maintenance. Their coexistence does not establish reciprocal causality. The sections below separate persistent molecular features from measured signaling and functional effects, identify the model supporting each claim, and indicate where a proposed connection remains inferential. **Figure 2** is a conceptual map of candidate interactions; selected feedback modules are supported experimentally, but a single causal network encompassing all layers has not been demonstrated.

**Figure 2:**
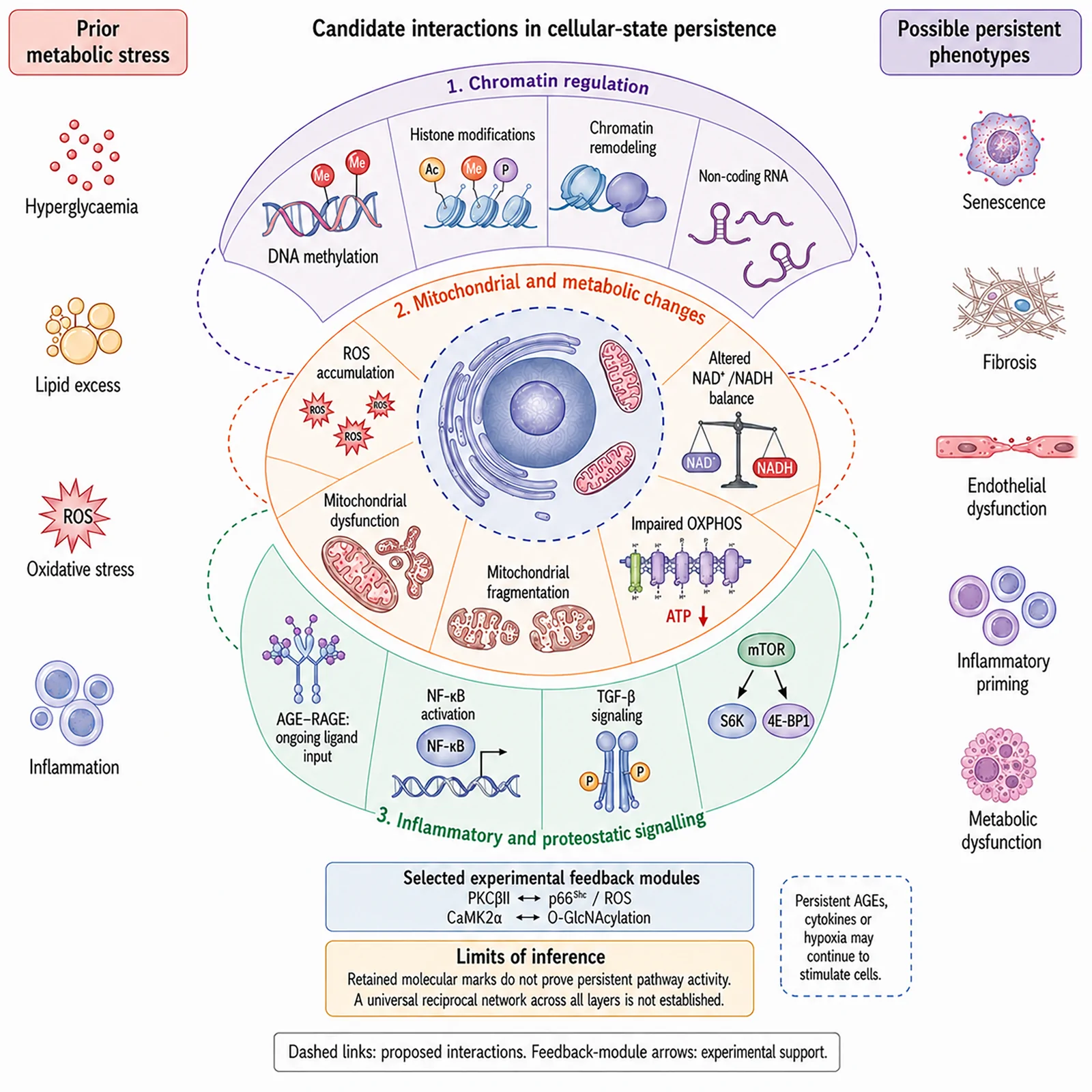
Candidate interactions involved in the encoding and maintenance of cellular states. Chromatin regulation, mitochondrial injury, metabolite availability and inflammatory or proteostatic signaling may interact. Direct post-normalization feedback evidence is confined to selected experimental modules, including PKCβII/p66Shc/ROS and CaMK2a/O-GlcNAcylation (34, 43). A universal reciprocal network incorporating every displayed layer has not been established. Continuing AGE, cytokine or hypoxic exposure is niche instruction unless independence from that input is tested. ECM, extracellular matrix; AGEs, advanced glycation end-products; ROS, reactive oxygen species; NF-κB, nuclear factor-κB; mTOR, mechanistic target of rapamycin.

### 3.1 Nuclear epigenetic memory

In human DCCT/EDIC blood samples, persistent methylation differences at TXNIP and other loci are associated with prior glycaemia and complications; locus-specific causal maintenance has not been shown in these participants (11). In human retinal endothelial cultures, DNMT activity, mitochondrial DNA methylation and MMP9-related hydroxymethylation persisted after four days at 20 mmol/L glucose followed by four days at 5 mmol/L glucose; extending recovery to eight days ameliorated selected abnormalities (16). Recovery-phase 5-aza-2′-deoxycytidine modified these endpoints, supporting a role for methylation machinery without isolating one causal CpG. Endothelial DNMT1/IMPDH2 experiments additionally link persistent methylation and nucleotide metabolism to excess sprouting; patient measurements in that study are associative (17). Methylation direction and the angiogenic phenotype are therefore locus- and model-dependent.

Controlled endothelial experiments link SETD7 recruitment and H3K4 methylation at RELA to persistent transcription and NF-κB target expression (9, 18). Perturbations preventing establishment do not by themselves prove that an already formed mark is required for maintenance. DCCT/EDIC monocyte H3K9 acetylation provides human association rather than a longitudinal demonstration of continuous NF-κB activity (10). In glucose-switch endothelial models, SIRT1/p300 manipulation modifies p53 acetylation, p21 and senescence (19). In mouse progenitor-derived macrophages, chromatin accessibility and RUNX1-associated programmes accompany heightened challenge responses and transferred atherosclerosis susceptibility (20). Accessibility or a primed enhancer alone does not establish ongoing kinase or transcription-factor activity.

The SETD7/RELA experiment illustrates the biological sequence more specifically. Human aortic endothelial cells were exposed to high glucose for 16 hours and then returned to normal-glucose culture for up to six days (9). SETD7 recruitment and histone H3 lysine 4 monomethylation at the RELA promoter accompanied increased expression of p65, a subunit of NF-κB. Increased CCL2/MCP-1 and VCAM1 expression connected the chromatin finding to inflammatory genes involved in monocyte recruitment and adhesion. SETD7 knockdown or reduction of mitochondrial superoxide-dependent events prevented the response. These results support glucose-induced chromatin regulation of persistent inflammatory gene expression. They do not show that delayed inhibition reverses an already established clinical vascular lesion. The accompanying mouse experiment used transient hyperglycemia in otherwise non-diabetic animals, rather than treatment of chronic diabetes.

Non-coding RNAs are candidate regulators when recovery-phase perturbation changes both signaling and function. Mouse and endothelial culture studies implicate miR-27a-3p/NRF2 and miR-29-associated oxidative or EndMT phenotypes (21). Experimental cardiac studies distinguish persistent miRNA profiles (22) from perturbation-based support for a miR-320/CD36 feedback module (23). Stable RNA abundance alone cannot distinguish active maintenance from slow decay or altered cell composition. These experiments do not establish the same RNA circuitry as causal in patients.

#### 3.1.1 Causal epigenetic maintenance versus retained marks

Causal interpretation is strongest when a recovery-phase perturbation changes a retained epigenetic feature together with its downstream activity and phenotype. Retinal DNMT inhibition (16), macrophage p66Shc methylation regulation (24), tubular DNMT1/p21 manipulation (25) and endothelial DNMT1/Ang-1 experiments (26) provide such mechanistic support at different levels. Broad DNMT or chromatin-enzyme manipulation remains pleiotropic and does not prove that a specific CpG or histone residue alone is necessary and sufficient. Human TXNIP methylation, monocyte acetylation and fibroblast methylation profiles remain primarily correlates (10, 11, 13). The distinction also applies to NRF2-mediated restoration of accessible chromatin, which demonstrates modifiability rather than locus-specific causality or treatment-free functional reset (27). Retinal SOD2 histone changes provide persistence and regulator-dependence evidence, but the cited induction-phase LSD1 intervention does not by itself establish delayed maintenance rescue (28).

### 3.2 Mitochondrial metabolic memory

In retinal animal and cell recovery models, mitochondrial DNA damage, impaired respiratory function and oxidant production can remain after glucose improvement (12, 29–33). Fission, TFAM handling and mitophagy are candidate contributors, but persistent organelle damage is not itself proof of an autonomous signaling loop. Stronger evidence comes from recovery-phase interventions that change ongoing oxidant production or organelle function. The vascular p66Shc model links persistent PKCβII-dependent activation, mitochondrial translocation and ROS generation to impaired nitric-oxide function, with rescue when p66Shc is silenced at glucose normalization (34). This supports a specific active redox mechanism in cultured cells and mice, not a universal mitochondrial-memory mechanism in humans.

In the p66Shc experiments, persistent PKCβII-dependent activation and mitochondrial localization of p66Shc were linked to continuing ROS production after glucose correction (34). ROS sustained PKCβII signaling, including inhibitory phosphorylation of endothelial nitric oxide synthase at Thr495, thereby connecting the pathway to reduced nitric oxide availability and impaired relaxation. Promoter hypomethylation and histone acetylation accompanied increased p66Shc expression. Silencing p66Shc at glucose normalization reduced ROS and improved endothelium-dependent relaxation. This functional response makes the mechanism more informative than a persistent marker alone. However, ongoing gene silencing does not establish how long normal function would persist after the perturbation ends. The related macrophage study linked activated protein C, protease-activated receptor 1 and restoration of DNMT1-dependent p66Shc promoter methylation to improvement in a proatherogenic phenotype (24), illustrating a cell-specific epigenetic intervention rather than a universal antioxidant treatment.

### 3.3 Metabolite–chromatin coupling

Acetyl-CoA, one-carbon metabolism and α-ketoglutarate-dependent enzymes provide established biochemical links between metabolism and chromatin (35–37), but those studies do not all examine diabetic exposure withdrawal. Persistent fumarate and mitochondrial abnormalities in treated diabetic rats are compatible with metabolite–epigenome coupling without proving that each component maintains the others (38). mTOR–HIF-1α-dependent glycolysis and mevalonate signaling are mechanistically supported in trained-immunity experiments, including β-glucan stimulation, mevalonate-associated training and oxidized-LDL exposure, rather than uniformly in post-normalization diabetic tissues (39–41). Convergence on these nodes is plausible; equivalence of initiating stimuli and maintenance mechanisms remains unproven.

### 3.4 Persistent signaling and proteostatic control

Direct post-normalization signaling measurements are available for selected mechanisms, not consistently across the literature. Examples include ROS-dependent glucose-stress persistence (42), vascular PKCβII/p66Shc activation (34), NF-κB transcriptional activity in glucose-switched cord-blood HSPCs (14), and endothelial CaMK2a activity in an O-GlcNAcylation feedback model (43). Transcript enrichment, DNA methylation and ATAC-seq accessibility should not be reported as kinase activation. AGE–RAGE signaling may instead reflect continued ligand exposure (15, 44). Autophagy and proteostasis remain relevant candidate modifiers, but much of their diabetic evidence comes from ongoing stress rather than controlled recovery (45).

### 3.5 Shared nodes do not establish a common maintenance network

Specific experimental feedback modules include p66Shc-derivedROS and PKCβII (34), miR-320/CD36 in the diabetic heart (23), and endothelial CaMK2a/O-GlcNAcylation (43). These support local reciprocal relationships, not causal integration of every proposed layer across organs. DNMT1/IMPDH2 inhibition provides another candidate coupling mechanism, but the human observations are associative (17). A network claim requires bidirectional maintenance-phase perturbation and rescue within the same system; simultaneous pathway changes can instead be parallel consequences of stress.

Stimulus specificity is demonstrable. In the macrophage study, hyperglycemia, but not hyperlipidemia, induced the p66Shc-associated phenotype (24). Lipid-training experiments support mevalonate and glycolytic pathways (40, 41); palmitate-withdrawal models link persistent insulin-signaling defects to FOXO1-associated histone changes without proving those marks causal (46). Conversely, most diet-induced hepatic transcriptional and H3K27ac changes recovered after weight loss in another mouse model (47). Hypoxia-related HIF signaling is not automatically memory if low oxygen persists, and inflammatory stimulation can produce either priming or other adaptive responses depending on context. No reviewed experiment establishes that glucose, lipid excess, hypoxia and inflammation converge on an identical self-sustaining circuit.

## 4 Cell-type-specific reprogramming

Shared memory mechanisms do not produce a uniform cellular endpoint. Their consequences are constrained by lineage identity, physiological function, regenerative capacity and tissue context (**Figure 3**). The same chromatin or redox pathway can therefore generate endothelial barrier failure, inflammatory progenitor bias, fibrosis, endocrine identity loss or defective epithelial repair. These trajectories are heterogeneous and partially overlapping rather than binary.

**Figure 3:**
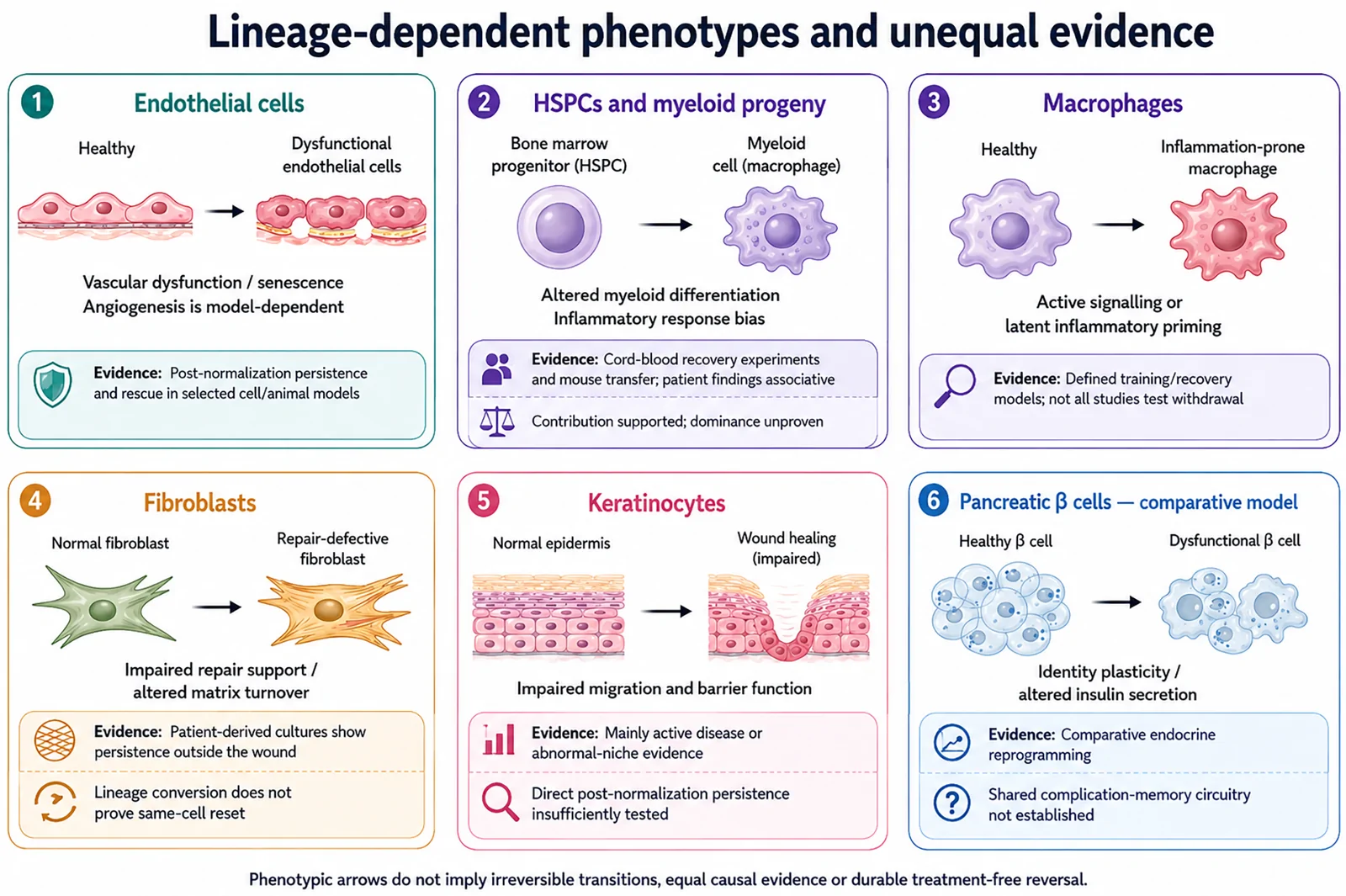
Lineage-dependent phenotypes and unequal evidence for cellular memory. Endothelial, HSPC, macrophage and fibroblast studies provide different degrees of post-normalization persistence and perturbation evidence. Keratinocyte observations predominantly reflect active disease or abnormal niches. β-cell identity plasticity is a comparative endocrine model, not an established manifestation of the same complication-memory circuitry. Phenotypic arrows do not imply equivalent causal evidence, irreversible transitions or a uniform angiogenic direction. **Table 2** specifies evidence and reversibility limits for each lineage.

### 4.1 Endothelial cells: dysfunction, senescence and partial EndMT

Endothelial dysfunction includes impaired nitric-oxide signaling, barrier disruption and abnormal angiogenesis. Acute eNOS and junctional studies establish responses during high glucose (48, 49); PGC-1α and SETD7–SEMA3G studies identify diabetes-associated angiogenic mechanisms without alone demonstrating durable post-withdrawal memory (50, 51). Direct persistence and intervention evidence comes from glucose-switch senescence, EndMT and p66Shc models (19, 21, 34, 52). The DNMT1/IMPDH2 study reports sustained excessive sprouting in its model (17), so memory should not be equated with uniformly reduced angiogenesis. Clinical causal validation of these maintenance mechanisms remains lacking.

### 4.2 Hematopoietic progenitors, monocytes, and macrophages

HSPCs provide one experimentally supported route for transmitting altered inflammatory responsiveness. In the Vinci study, cord-blood CD34-positive cells underwent high-glucose exposure followed by normoglycemic recovery and differentiation; patient bone-marrow HSPCs showed a concordant disease-associated signature but were not a longitudinal normalization experiment (14). In mice, diabetic marrow transferred increased atherosclerosis susceptibility to normoglycemic Ldlr-deficient recipients (20), and HSC experiments linked DNMT1-dependent differentiation defects to impaired wound repair (53). These results support transferability in specified models. Whole-marrow transplantation does not isolate every HSPC contribution, and irradiation, engraftment and recipient atherogenic conditions affect interpretation. Neither experiment establishes that HSPCs are the quantitatively dominant driver in all diabetic organs or in humans.

The functional meaning of trained immunity is an altered later response. Macrophages from diabetic mice could retain abnormal responses at physiological glucose, and diabetic marrow transferred increased atherosclerosis susceptibility to normoglycemic Ldlr-deficient recipients (20). RUNX1-associated chromatin findings and pharmacological inhibition in culture provide a candidate regulatory connection. Whole-marrow transplantation, conditioning and the recipient atherogenic environment limit attribution to a single progenitor population. These results should therefore not be equated with proof that all persistent inflammation in a treated patient is inherited from bone marrow.

Mature monocytes and macrophages can also acquire response bias independently of progenitors. High-glucose training experiments show exaggerated secondary responses (54), while intermittent hyperglycemia and lipid or microbial training engage partially overlapping myelopoietic and metabolic pathways (39–41, 55). These designs do not all test maintenance after complete stimulus withdrawal. In wounds and kidneys, necrotic material, hypoxia and local cytokines can continuously reactivate arriving cells. Relative contributions from progenitor programming, resident macrophages and niche instruction require matched lineage-specific interventions; a fixed M1/M2 classification does not resolve them.

### 4.3 Fibroblasts: fibrotic and repair-defective states

Patient-derived diabetic foot ulcer fibroblasts retain methylation abnormalities during culture and impaired support functions in reconstructed skin (13, 56). This supports persistence outside the original wound, although donor comorbidity and culture selection limit attribution to glucose alone. Pluripotency-based reprogramming and vasculogenic conversion demonstrate plasticity (57, 58), not necessarily durable restoration of the same mature fibroblast identity. YAP/TAZ experiments in fibrosis support mechanosensitive activation, but the cited study is not a diabetic kidney or heart washout experiment (59). In wounds, TNF-α/SETDB2 can suppress fibroblast-to-myofibroblast transition during active disease (60); continued cytokine exposure should be separated from autonomous fibroblast memory.

### 4.4 Pancreatic β cells as a comparative endocrine model

β cells are included to illustrate recovery and identity plasticity, not as another diabetic complication or proof of a shared vascular-memory circuit. PDX1, MAFA, NKX6.1 and FOXO1 coordinate mature identity and mitochondrial stimulus–secretion coupling (61–66). Human pancreatic studies identify disease-associated identity changes, but they do not establish persistence after verified normalization (61, 65, 67). Rodent lineage tracing and metabolic-relief experiments demonstrate dedifferentiation and possible redifferentiation; ER-stress models show adaptation and reduced recovery with repeated stress (62, 68–70). Senolysis can improve experimental β-cell function (71), but cell depletion, reversible glucotoxicity and lineage reprogramming are distinct endpoints. Shared oxidative or epigenetic features do not establish that β-cell dysfunction uses the same SETD7/NF-κB, HSPC or endothelial p66Shc maintenance circuitry.

### 4.5 Keratinocytes: failure to re-enter a regenerative state

Keratinocyte studies largely describe active diabetic wounds or ongoing high-glucose exposure. Wound-edge migration and differentiation defects, altered FOXO1 targets, impaired autophagy and inflammatory crosstalk are supported in those settings (72–79). Single-cell wound atlases and CXCR2/senescence studies identify abnormal states and niches without independently verifying post-normalization cellular persistence (80–82). We therefore classify keratinocyte-specific memory as insufficiently established. Their dysfunction may be continuously instructed by immune cells, perfusion deficits and matrix abnormalities; modifiability during diabetes does not demonstrate a sustained recovery after treatment cessation.

**Table 2** summarizes lineage-specific phenotypes together with the directness of post-normalization evidence, measured outputs and limits of reversibility. No uniform cellular endpoint or common strength of evidence is assumed.

## 5 Persistent dysfunction across diabetic complications

Each organ can contain three coexisting components: candidate cellular persistence after exposure withdrawal, continued pathological stimulation from the niche, and irreversible or slowly reversible structural damage. The following sections apply this distinction explicitly. Whole-organ dysfunction or current normoglycemia alone cannot identify the relative contribution of these components (**Figure 4**).

**Figure 4:**
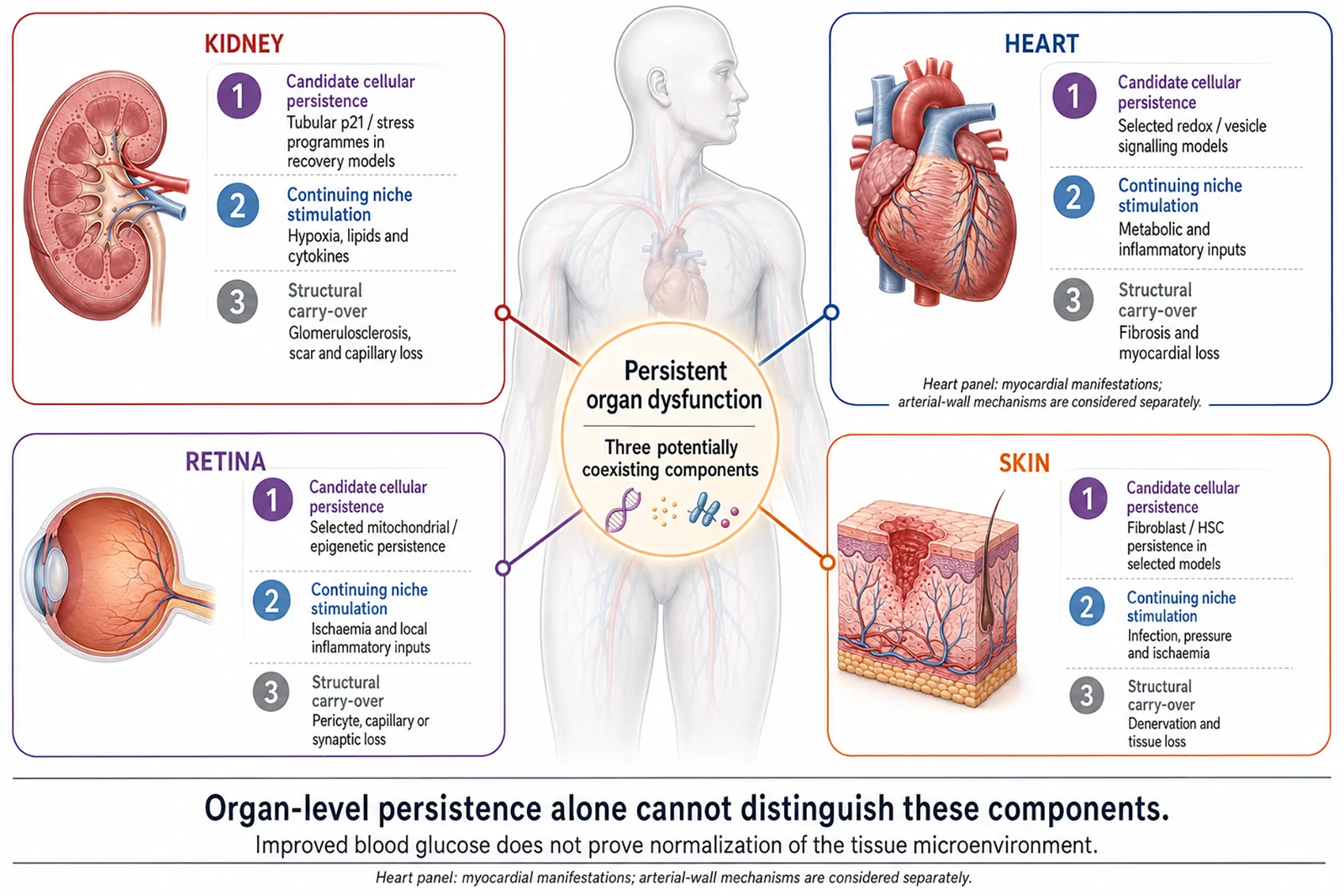
Components of persistent organ dysfunction in diabetes. Kidney, cardiovascular, retinal and wound disease can contain candidate cellular memory, continued pathological niche stimulation and persisting tissue damage. Hypoxia, plaque lipids, infection and matrix signals may remain active after glucose lowering; scar, capillary loss and denervation can outlast the initiating exposure without autonomous signaling memory. The cardiac panel concerns myocardial manifestations, whereas arterial-wall mechanisms are discussed separately in Section 5.2. Organ-level persistence alone cannot distinguish these components.

### 5.1 Diabetic kidney disease: glomerular, tubular, and stromal mismatch

Post-correction cellular evidence in diabetic kidney disease is mainly experimental. Podocyte SHP-1, endothelial Sirt7-associated programmes and tubular p21 persist in animal or cell glucose-recovery models (25, 52, 83, 84). The p21 study also reports human tissue or urinary associations and persistence despite improved glycaemia; the DNMT1-dependent causal interventions are preclinical, not performed in patients (25). Tubular lipid accumulation in human biopsies and spatial fibrotic signatures identify disease-associated biology, not autonomous memory after normalization (85, 86). Continued tubular workload, lipids, hypoxia and cytokines constitute niche stimulation. Glomerulosclerosis, interstitial scar and capillary loss constitute persisting tissue damage. These processes can coexist, and even the p21 study cannot fully exclude glomerular-to-tubular instruction *in vivo* (25).

Mechanistically, the tubular study linked reduced DNA methyltransferase 1 (DNMT1) to hypomethylation of the CDKN1A promoter and sustained expression of its product, the cell-cycle inhibitor p21 (25). DNMT1 suppression in tubular cells increased p21. In diabetic mice, adding activated protein C to glucose-lowering treatment restored DNMT1 and promoter methylation and improved tubular injury measures; interfering with DNMT1 weakened the benefit. This supports a regulatory contribution to persisting injury in the experimental system. Increased p21 alone is not sufficient to diagnose senescence, because it also participates in the DNA-damage response. Human tissue and urinary observations support clinical relevance but do not demonstrate treatment causality. The direction of methylation change matters: restoring DNMT1-associated repression in this model differs from inhibiting increased methylation activity in retinal models (16). A general treatment intended to remove DNA methylation would therefore obscure potentially opposite effects at different loci.

### 5.2 Cardiovascular complications: vascular and myocardial memory

Vascular cellular persistence is supported by endothelial p66Shc experiments and diabetic marrow transfer in mice (20, 34). Recurrent hyperglycemia-induced myelopoiesis is related evidence but does not remove repeated exposure as an explanation (55). Plaque lipids and AGE-modified matrix remain local stimuli; established atheroma and myocardial scar are structural disease. In the myocardium, animal studies support persistent miRNA changes and a miR-320/CD36 feedback module after glycemic correction (22, 23). Experimental endothelial vesicle transfer provides evidence for a circulating source of continued cardiac injury, whereas the patient vesicle measurements are associations (43). Recent aortic single-cell data show persistent contractile and chromatin abnormalities with partial improvement of other programmes after dapagliflozin; these are molecular persistence findings, not direct proof of continuous activity of every enriched pathway (87). **Figure 4** distinguishes vascular and myocardial manifestations.

### 5.3 Diabetic retinopathy: persistent neurovascular-unit dysfunction

Retinal recovery models support persistent mitochondrial injury, methylation-related changes and abnormal organelle quality control (12, 16, 29–33, 88). Evidence is strongest in animal retinas and controlled retinal cell cultures; equivalent causal maintenance has not been established in patients. Ischemic VEGF drive, gliosis and inflammation may reflect ongoing local stimuli (89–91). Pericyte loss, non-perfused capillaries and synaptic loss represent structural or slowly reversible damage. Persistence is also endpoint- and time-dependent: longer normal-glucose recovery ameliorated selected methylation abnormalities (16), and delayed insulin normalized several retinal inflammatory measures while synapse-loss markers remained abnormal (92). Residual retinal disease therefore does not imply universal ongoing inflammatory signaling.

A complementary retinal study examined MFN2, involved in mitochondrial fusion, and MLH1, involved in DNA repair (136). Retinal endothelial cells were exposed to 20 mmol/L glucose for four days followed by 5 mmol/L for four days; diabetic rats underwent poor control followed by an extended period of improved glycaemia. Persistent methylation abnormalities accompanied impaired retinal function and continuing retinopathy. These findings connect particular gene-regulatory abnormalities to mitochondrial stability, while leaving their causal contribution in patients unresolved. Together with partial recovery in the earlier DNMT study (16), they show why the affected gene, functional endpoint and recovery interval must all be specified.

### 5.4 Diabetic foot ulcers: failed multicellular wound-state resolution

In diabetic foot disease, ex vivo fibroblast persistence and HSC transfer models support selected cellular contributions (13, 53, 56), while keratinocyte-specific post-normalization evidence remains limited. Endothelial DNMT1/Ang-1 experiments add preclinical support for persistent vascular dysfunction and impaired healing (26). Infection, wound biofilm, ischemia, pressure and inflammatory matrix signals are continuing stimuli; neuropathy, arterial disease and tissue loss provide structural vulnerability. Wound-microbiome associations do not establish autonomous host-cell memory (93, 94). Post-closure barrier leakage predicts recurrence (95), but neither incomplete barrier restoration nor recurrence identifies a specific memory circuit. Offloading and temperature-guided prevention address continuing mechanical injury and remain relevant independently of cellular-memory hypotheses (96, 97).

## 6 From cell-autonomous memory to tissue and inter-organ memory

### 6.1 Cell-autonomous memory

Cell-autonomous persistence is supported when previously exposed cells remain abnormal in a common, controlled environment, as in fibroblast culture, progenitor differentiation and endothelial glucose-switch experiments (13, 14, 19–21, 52, 53, 56). This inference is strengthened by serial passage, defined medium and a healthy matrix, with matched cell density, oxygen, lipids and cytokines. Reciprocal experiments should place exposed cells in a healthy niche and naïve cells in a disease-associated niche. Conditioned-medium transfer, matrix replacement and receptor blockade can identify continued extracellular instruction; donor-state persistence after these manipulations supports an intrinsic component. These are discriminating designs, not experiments already completed across all lineages. Residual ligands, clonal selection and newly produced autocrine factors must still be measured.

### 6.2 Niche and extracellular-matrix memory

Long-lived AGE-modified collagen and matrix stiffness can continually stimulate otherwise naïve cells through adhesion, mechanosensing and TGF-β-related mechanisms (15, 59, 98). Hypoxia, denervation, cell loss and senescent-cell secretions may reinforce the same tissue phenotype (82). We use niche-dependent persistence for ongoing local instruction and persisting tissue damage for slow turnover or tissue loss. Neither term is interchangeable with cell-autonomous metabolic memory. A matrix may store consequences of previous exposure, but signaling elicited by that persisting matrix remains an active environmental input.

### 6.3 The bone marrow–organ axis

The bone marrow–organ axis is a candidate propagation route supported particularly by mouse atherosclerosis and wound models (20, 53). Human HSPC signatures and experimental differentiation provide biological plausibility (14), while recurrent hyperglycemia-driven myelopoiesis demonstrates another, exposure-dependent process (55). Necessity, sufficiency and the fraction of persistent organ inflammation attributable to HSPCs have not been established across kidney, retina and myocardium. Selective progenitor manipulation must be compared with resident-cell and niche interventions before assigning dominance.

### 6.4 Extracellular vesicles and circulating mediators

In mouse and endothelial experiments, sustained CaMK2a/O-GlcNAcylation signaling promotes continued vesicular miR-15/16 release after glucose correction and transfers cardiac dysfunction to recipients (43). This supports a specific signaling and intercellular propagation module. The associated human vesicle measurements do not prove that the module causally maintains dysfunction after documented metabolic normalization. Recipient cardiomyocytes receiving persistent vesicle input are still exposed to a pathological signal; a separate recipient washout experiment is needed before calling their response autonomous memory.

### 6.5 Microbiome contributions and unresolved persistence

The reviewed evidence does not establish that diabetic gut or wound microbiome changes persist after verified metabolic normalization and independently maintain host pathological signaling. Hyperglycemia-related barrier dysfunction (99), Western-diet/NLRP3-dependent immune training (100), imidazole-propionate effects on insulin signaling (101) and wound-community associations (93, 94) address different questions. The diet study does not isolate the microbiome as the carrier, and a microbial metabolite acting while present is continuing exposure. A causal test would require longitudinal microbial and metabolite measurements after correction, matched diet and medication controls, transfer to metabolically matched recipients, and loss/rescue experiments. Durable host priming after a microbial signal has cleared would demonstrate host memory, not necessarily persistent microbial dysbiosis.

The current evidence supports coexistence of acquired cellular states, continued niche instruction and structural injury. Their relative contributions remain unresolved; the broader distributed-network interpretation is a hypothesis to test rather than a finding common to all organs.

## 7 Prevention state modification and durable resetting

We use three therapeutic categories. Prevention limits exposure or blocks encoding before persistence is established. State modification changes an established molecular or functional phenotype; suppression denotes reduced output during treatment. Sustained molecular and functional recovery after treatment cessation requires restoration of both state-associated biology and function that remains after the intervention and its pharmacological effects have cleared, ideally with a normalized response to rechallenge. Reversal refers only to the specific measured endpoint unless treatment-free durability is demonstrated. Reprogramming describes a change of cellular programme or identity and does not itself establish therapeutic benefit. **Figure 5** and **Table 3** use these definitions.

**Figure 5:**
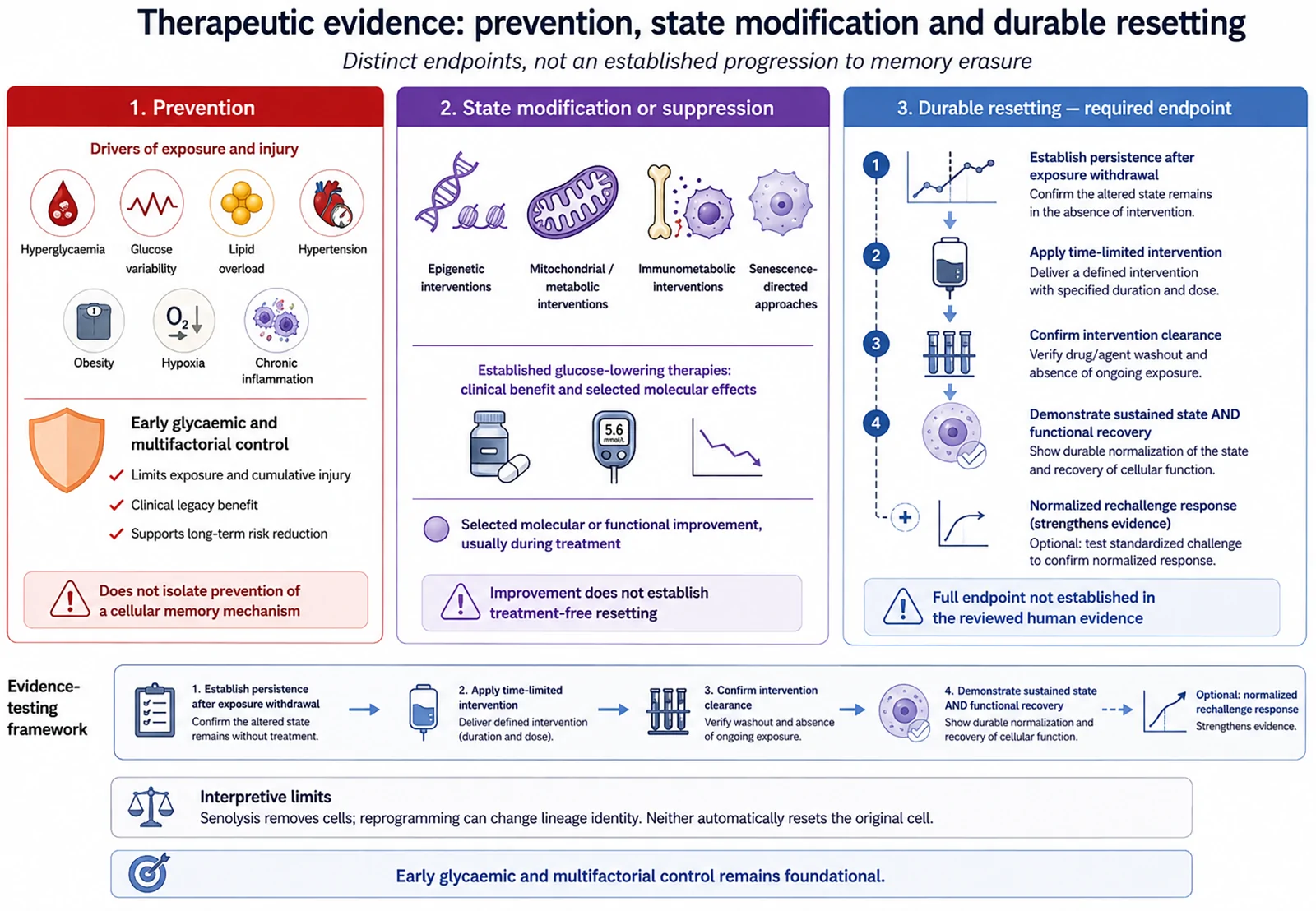
Therapeutic evidence organized by endpoint. Prevention limits exposure or encoding; state modification or suppression changes established abnormalities, usually assessed during treatment; sustained molecular and functional recovery after treatment cessation requires persistent molecular and functional recovery after intervention clearance, ideally with normalized rechallenge responses. Experimental epigenetic, mitochondrial, immunometabolic and senescence-directed approaches mainly occupy the second category. Senolysis removes cells and lineage reprogramming changes identity, so neither automatically demonstrates reset of the original cell. Durable clinical resetting of the reviewed signaling states remains unestablished.

**Table 3:** Prevention state modification and the evidence gap for sustained molecular and functional recovery after treatment cessation.

Therapeutic category	Intervention and target	Evidence and demonstrated endpoint	After intervention withdrawal	Interpretive limit
Prevention	Early glycemic and multifactorial management (1–6, 102, 103).	Clinical reduction of later events; reduced cumulative injury.	Legacy benefit after the randomized treatment phase.	Not a demonstration that a specific cellular memory was formed or erased.
Prevention or state modification	SETD7/SIRT1/p300 and trained-immunity metabolic interventions (19, 39–41, 51).	Cell/animal encoding inhibition or intervention-associated molecular/functional improvement, depending on timing.	NR for full diabetic signaling and functional reset.	Induction blockade must not be described as reversal of established memory.
State modification	Retinal DNMT modulation; macrophage p66Shc and tubular p21 regulation through aPC-related signaling (16, 24, 25).	Preclinical recovery-phase modification of methylation-related endpoints, stress outputs and selected functions.	NR.	DNMT direction is locus-specific; pleiotropy and continuing treatment limit reset inference.
State modification	DNMT1/IMPDH2 or DNMT1/Ang-1; NRF2/sulforaphane (17, 26, 27).	Experimental changes in persistent angiogenesis, inflammatory or chromatin/transcriptional abnormalities.	NR.	Excess sprouting and poor angiogenesis are different model phenotypes. Molecular recovery does not ensure durable functional reset.
State modification	p66Shc, mitochondrial fission, TFAM and mitophagy interventions (29–34).	Experimental rescue of persistent organelle/redox abnormalities and selected functions.	NR.	Persistent gene silencing or target suppression cannot test an intervention-free state.
Protection or state modification	NAD precursors and ketone/NRF2-associated interventions (105, 106).	Predominantly preclinical protection or on-treatment changes during diabetes.	NR.	Exposure-normalization and delayed-intervention criteria are not uniformly met.
Cell depletion or output suppression	Senolytics and senomorphics (71, 108–112).	Animal efficacy; early human target engagement or clinical outcomes.	Some post-dose observations occur, but a paired memory-state/function reset is not established.	Cell removal differs from resetting the original cells; sustained effect alone is insufficient.
Cellular reprogramming	Pluripotency or vasculogenic fibroblast conversion (57, 58).	Experimental plasticity and reparative outputs.	A durable change of identity is not equivalent to restoration of the same lineage.	Cannot infer erasure of all diabetic-memory mechanisms.
Clinical disease modification	Insulin, metformin, SGLT2 inhibitors, GLP-1 receptor agonists, pioglitazone (19, 41, 92, 114–119).	Clinical outcome benefit and selected molecular effects during treatment.	NR for directly measured diabetic memory-state plus function.	Maintain indicated treatment; outcome trials do not isolate reset mechanisms.
Experimental platform	Locus-specific dCas9 methylation editing (113).	*In vivo* technology proof of concept outside validated diabetic-memory resetting.	Not demonstrated for diabetic memory.	Delivery, target specificity and safety require validation.
Sustained molecular and functional recovery after treatment cessation	Time-limited treatment after verified memory formation, followed by intervention clearance and functional follow-up.	Required experimental endpoint; no reviewed human strategy satisfies the full criteria.	Must be directly demonstrated; rechallenge strengthens inference.	An evidence gap, not an established therapeutic class.

Prevention, on-treatment state modification and recovery sustained after experimental treatment cessationting are separate endpoints. NR means that an adequate intervention-withdrawal assessment was not demonstrated in the evidence considered, not proof that durability is impossible. Permanent genetic manipulation and senescent-cell depletion do not by themselves show reset of the same surviving cells.

### 7.1 Prevention of memory formation and cumulative injury

Early intensive glycemic and multifactorial management provides the strongest clinical evidence for reducing later complications (1–6, 102). These trials support prevention of cumulative injury and clinical legacy benefit; they do not isolate prevention of a particular cellular memory mechanism. Glucose variability studies and experimental early-retinopathy responses support attention to exposure dynamics, but do not establish sustained recovery after experimental treatment cessation (103, 104). Conventional management remains foundational regardless of which persistence mechanism operates.

### 7.2 Modification or suppression of established abnormalities

Epigenetic interventions provide selected preclinical evidence of state modification. Recovery-phase DNMT inhibition changes retinal methylation-related endpoints (16), whereas aPC-associated restoration of DNMT1 modifies macrophage p66Shc and tubular p21 programmes (24, 25). This opposite direction of DNMT manipulation argues against a universal methylation-erasing treatment. DNMT1/Ang-1 work links an epigenetic intervention to endothelial signaling and healing (26). NRF2 activation with sulforaphane can restore transcription and chromatin accessibility in human endothelial cultures exposed to 30 mmol/L glucose for four days followed by 5.5 mmol/L glucose for four days (27). These experiments support modifiability at measured endpoints; they do not establish long-term treatment-free functional reset in patients.

#### 7.2.1 Mitochondrial and metabolic interventions

p66Shc silencing at glucose normalization improves experimental vascular function (34). Retinal fission, TFAM and mitophagy interventions address continuing organelle injury in selected recovery models (29–33). NAD precursors and SGLT2-associated ketone/NRF2 effects mainly provide protection or state modification during treatment (105, 106). Persistent inhibitor exposure or genetic knockdown cannot demonstrate treatment-free durability. Candidate mitochondrial therapies also require a distinction between replacing damaged organelles and eliminating a mechanism that would damage replacements.

#### 7.2.2 Immunometabolic interventions

mTOR–HIF-1α, mevalonate and glucose-metabolic interventions can block induction of trained immunity in defined systems (39–41). Their ability to erase established diabetic training is less certain. Statin-treated familial hypercholesterolemia provides a non-diabetic lipid-exposure comparison in which the reported proinflammatory phenotype was not reverted (107); it should not be taken as proof that every diabetic memory resists lipid lowering. HSPC-directed approaches remain experimental, and intervention must preserve antimicrobial defense and balanced hematopoiesis. Promotion of efferocytosis is biologically plausible, but comparative safety or sustained recovery after experimental treatment cessation has not been established in this setting.

#### 7.2.3 Senescence and cellular reprogramming

Senolytics remove susceptible cells, whereas senomorphics suppress selected senescence-associated outputs. Experimental β-cell and wound studies, and early clinical studies in diabetic kidney disease and macular oedema, provide evidence for target engagement or disease modification (71, 108–112). Cell clearance with a lasting effect is not equivalent to restoration of the original cells. Pluripotency-based or vasculogenic reprogramming likewise demonstrates plasticity but may change lineage identity (57, 58). SETD7 inhibition and *in vivo* epigenome-editing platforms are mechanistic or technological advances; neither is established as durable clinical diabetic-memory resetting (51, 113).

#### 7.2.4 Established glucose-lowering therapies

SGLT2 inhibitors and GLP-1 receptor agonists reduce clinical cardiorenal outcomes (114–116). Molecular studies of SGLT2 inhibition, incretin drugs, metformin and pioglitazone show varying degrees of state modification or prevention (19, 41, 106, 117–119), but clinical benefit does not identify an erased memory mechanism. Insulin can normalize retinal inflammatory measures while synaptic abnormalities remain (92). None of these observations demonstrates that normalization of a memory-associated signaling state and organ function both persist after drug withdrawal. The limits of that endpoint are summarized in Section 7.3 and **Table 3**.

### 7.3 Sustained molecular and functional recovery after treatment cessation after intervention withdrawal

Among the selected studies, none establishes durable reversal of the full underlying signaling and functional state after treatment withdrawal in patients with diabetic complications. Experimental recovery-phase rescue with p66Shc silencing, aPC-related signaling, DNMT modulation or NRF2 activation supports state modification, but an adequately documented intervention-free functional follow-up and standardized rechallenge are absent from the evidence used to make these claims (16, 24, 25, 27, 34). The word reversal in a study title does not establish this stricter endpoint.

A decisive design first establishes persistence under normalized exposure, applies a time-limited intervention, confirms clearance or termination of target perturbation, and follows the same lineage for recovery of molecular activity and function. Rechallenge tests whether response bias has normalized. Continued glucose control is a necessary background condition, not a reason to withdraw clinically indicated therapy in patients; such tests may first be conducted in preclinical or ex vivo systems. Durability must not be inferred from ongoing drug action, permanent gene silencing, cell replacement or lasting tissue benefit alone.

## 8 Technologies needed to map and test metabolic memory

### 8.1 Longitudinal single-cell and spatial multi-omics

Single-time-point omics identifies disease-associated states but cannot prove memory. A rigorous design samples the encoding phase, early and late normalization, and recurrence or rechallenge. Longitudinal single-cell RNA sequencing should be paired with chromatin accessibility, DNA methylation, metabolomics and spatial context. Existing diabetic foot and kidney atlases demonstrate extensive cellular heterogeneity and fibrotic or inflammatory niches, but cross-sectional pseudotime is not elapsed biological time (80, 81, 86). Joint RNA–chromatin profiling can reveal cells whose basal transcriptome appears normalized while enhancers remain primed (120), and single-cell methylome approaches can link methylation to transcription within the same lineage (121). Spatial transcriptomics and metabolomics are required to distinguish cell-autonomous memory from matrix, hypoxia or regional metabolic instruction (86, 122).

### 8.2 Lineage tracing and cellular barcoding

Lineage tracing and cellular barcoding determine whether a pathological population consists of the same long-lived cells, descendants of a reprogrammed progenitor or newly recruited cells instructed by an abnormal niche. Ms4a3 fate mapping distinguishes monocyte-derived from resident macrophages (123), while CellTag and CRISPR recorder systems link ancestry to transcriptomic state (124, 125). Applied before metabolic exposure and after normalization, these approaches could identify clones that recover, remain trapped or reactivate after challenge.

### 8.3 Perturbation-based validation

Perturbations should be timed separately to induction and maintenance. Perturb-seq and multiome approaches can identify candidate regulators (126, 127), while locus-specific epigenome editing can test the necessity and sufficiency of a defined lesion (113). Network causality additionally requires reciprocal perturbation, temporal ordering and rescue or epistasis within the same recovery system; pathway enrichment or correlated nodes cannot supply this evidence. Organoids and glomerulus-on-chip models provide human experimental contexts, but become memory models only with documented exposure withdrawal, repeated recovery measurements and functional endpoints (128, 129).

### 8.4 Clinically measurable memory biomarkers

Clinically useful biomarkers must capture historical exposure or persistent biology beyond current glycated hemoglobin and must improve a treatment or surveillance decision. Candidate measures include blood DNA methylation, extracellular-vesicle microRNAs, functional trained-monocyte responses, SASP panels, mitochondrial metabolites, skin autofluorescence, retinal optical coherence tomography angiography and post-closure transepidermal water loss (43, 95, 130–133). No validated metabolic-memory score is available. A future score will probably require three layers: cumulative exposure history, evidence of persistent systemic or cellular reprogramming, and an organ-specific measure of residual vulnerability.

## 9 Outstanding questions

The major unresolved issues are summarized in **Box 2**. They concern state continuity, adaptive versus pathogenic reprogramming, organ-specific thresholds, inheritance through progenitors or tissue renewal, separation of glucose-, lipid-, hypoxia- and inflammation-induced memory, and the criteria by which a therapy can be said to reset rather than suppress a pathological state.

Box 2 **Outstanding questions for the field.**

1. Is metabolic memory a set of discrete states or a continuous spectrum of response bias and reduced plasticity?
2. Which stress-associated states are adaptive, and which become causally pathogenic when they persist?
3. Does memory establishment have a therapeutic window beyond which glycemic normalization alone becomes insufficient?
4. Do kidney, retina, heart and skin have different exposure thresholds and recovery capacities?
5. What proportion of multiorgan persistence is generated by trained HSPCs and their myeloid descendants?
6. Can memories induced by glucose, lipids, hypoxia, inflammation and microbial products be mechanistically distinguished?
7. Can memory persist through tissue renewal, and is it transmitted by progenitors, extracellular matrix or repeated niche instruction?
8. Which therapies durably reset a state after withdrawal rather than suppressing damage markers while treatment continues?
9. How should cumulative exposure, systemic memory and organ-specific vulnerability be combined for patient stratification?
10. Does biochemical target attainment or clinical wound closure indicate restoration of tissue homeostasis?

## 10 Discussion and perspective

The evidence supports selected cellular memory phenotypes but does not justify relabeling all persistent diabetic injury as an actively maintained memory state. The operational definition requires verified exposure withdrawal and a history-dependent functional phenotype; causal maintenance and sustained recovery after the experimental treatment ends require additional experiments. Longitudinal clinical legacy effects, persistent blood epigenetic marks and experimental signaling loops answer different questions.

The strongest mechanistic examples link retained molecular changes to measured activity and intervention-sensitive function in defined animal or cell models. Other observations establish only molecular persistence, continuing local inflammatory, hypoxic or matrix-derived stimulation or persisting tissue damage. Active signaling and latent challenge responsiveness should be reported separately. Human causal evidence remains limited, and broad claims of reciprocal reinforcement among all epigenetic, mitochondrial, metabolic, inflammatory and microenvironmental layers exceed the available data.

HSPC training is a plausible contributor to persistent inflammation, supported by transfer and differentiation experiments, but its dominance over resident-cell programmes and niche signals has not been established. β-cell identity changes, microbial influences and lipid-induced training offer informative comparisons without proving one universal diabetic-memory circuit. The working framework is useful only if it can accommodate recovery, stimulus specificity and negative findings.

Clinically, early glycemic and multifactorial control remains the best-supported means of limiting later complications. Experimental state modification identifies candidate maintenance mechanisms but should not be presented as established recovery sustained after experimental treatment cessation. No intervention in the reviewed human evidence demonstrates the full combination of verified normalization, reversal of the memory-associated signaling state, functional recovery and durability after intervention withdrawal. Structural injury may independently limit organ recovery even if a cellular state is modified.

Future studies should combine exposure history, lineage information, direct activity assays and post-normalization perturbation, with follow-up after intervention clearance. Their purpose is to discriminate acquired cellular persistence from environmental re-instruction and lasting damage, and to quantify the contribution of each. The narrative selection and targeted source appraisal used here do not establish the prevalence of persistence across all studies; the proposed framework remains testable rather than validated as a clinical diagnostic system.

## References

[1] Diabetes Control and Complications Trial Research Group . The effect of intensive treatment of diabetes on the development and progression of long-term complications in insulin-dependent diabetes mellitus. N Engl J Med. (1993) 329:977–86. doi: 10.1056/NEJM199309303291401 8366922

[2] Writing Team for the Diabetes Control and Complications Trial/Epidemiology of Diabetes Interventions and Complications Research Group . Sustained effect of intensive treatment of type 1 diabetes mellitus on development and progression of diabetic nephropathy: the EDIC study. JAMA. (2003) 290:2159–67. doi: 10.1001/jama.290.16.2159 14570951 PMC2622725

[3] White NH Sun W Cleary PA Danis RP Davis MD Hainsworth DP . Prolonged effect of intensive therapy on the risk of retinopathy complications in patients with type 1 diabetes mellitus: 10 years after the Diabetes Control and Complications Trial. Arch Ophthalmol. (2008) 126:1707–15. doi: 10.1001/archopht.126.12.1707 19064853 PMC2663518

[4] Diabetes Control and Complications Trial/Epidemiology of Diabetes Interventions and Complications Study Research Group . Intensive diabetes treatment and cardiovascular outcomes in type 1 diabetes: the DCCT/EDIC Study 30-year follow-up. Diabetes Care. (2016) 39:686–93. doi: 10.2337/dc15-1990 26861924 PMC4839174

[5] Holman RR Paul SK Bethel MA Matthews DR Neil HAW . 10-year follow-up of intensive glucose control in type 2 diabetes. N Engl J Med. (2008) 359:1577–89. doi: 10.1056/NEJMoa0806470 18784090

[6] Adler AI Coleman RL Leal J Whiteley WN Clarke P Holman RR . Post-trial monitoring of a randomised controlled trial of intensive glycaemic control in type 2 diabetes extended from 10 years to 24 years (UKPDS 91). Lancet. (2024) 404:145–55. doi: 10.1016/S0140-6736(24)00537-3 38772405

[7] Laiteerapong N Ham SA Gao Y Moffet HH Liu JY Huang ES . The legacy effect in type 2 diabetes: impact of early glycemic control on future complications. Diabetes Care. (2019) 42:416–26. doi: 10.2337/dc17-1144 30104301 PMC6385699

[8] Cardoso CRL Leite NC Moram CBM Salles GF . Long-term visit-to-visit glycemic variability as predictor of micro- and macrovascular complications in patients with type 2 diabetes: the Rio de Janeiro Type 2 Diabetes Cohort Study. Cardiovasc Diabetol. (2018) 17:33. doi: 10.1186/s12933-018-0677-0 29477146 PMC6389075

[9] El-Osta A Brasacchio D Yao D Pocai A Jones PL Roeder RG . Transient high glucose causes persistent epigenetic changes and altered gene expression during subsequent normoglycemia. J Exp Med. (2008) 205:2409–17. doi: 10.1084/jem.20081188 18809715 PMC2556800

[10] Miao F Chen Z Genuth S Paterson A Zhang L Wu X . Evaluating the role of epigenetic histone modifications in the metabolic memory of type 1 diabetes. Diabetes. (2014) 63:1748–62. doi: 10.2337/db13-1251 24458354 PMC3994951

[11] Chen Z Miao F Paterson AD Lachin JM Zhang L Schones DE . Epigenomic profiling reveals an association between persistence of DNA methylation and metabolic memory in the DCCT/EDIC type 1 diabetes cohort. Proc Natl Acad Sci USA. (2016) 113:E3002–11. doi: 10.1073/pnas.1603712113 27162351 PMC4890596

[12] Madsen-Bouterse SA Mohammad G Kanwar M Kowluru RA . Role of mitochondrial DNA damage in the development of diabetic retinopathy, and the metabolic memory phenomenon associated with its progression. Antioxid Redox Signal. (2010) 13:797–805. doi: 10.1089/ars.2009.2932 20088705 PMC2935337

[13] Park LK Maione AG Smith A Gerami-Naini B Iyer LK Mooney DJ . Genome-wide DNA methylation analysis identifies a metabolic memory profile in patient-derived diabetic foot ulcer fibroblasts. Epigenetics. (2014) 9:1339–49. doi: 10.4161/15592294.2014.967584 25437049 PMC4622843

[14] Vinci MC Costantino S Damiano G Rurali E Rinaldi R Vigorelli V . Persistent epigenetic signals propel a senescence-associated secretory phenotype and trained innate immunity in CD34-positive hematopoietic stem cells from diabetic patients. Cardiovasc Diabetol. (2024) 23:107. doi: 10.1186/s12933-024-02195-1 38553774 PMC10981360

[15] Lyons TJ Bailie KE Dyer DG Dunn JA Baynes JW . Decrease in skin collagen glycation with improved glycemic control in patients with insulin-dependent diabetes mellitus. J Clin Invest. (1991) 87:1910–5. doi: 10.1172/JCI115216 1904067 PMC296942

[16] Mishra M Kowluru RA . The role of DNA methylation in the metabolic memory phenomenon associated with the continued progression of diabetic retinopathy. Invest Ophthalmol Vis Sci. (2016) 57:5748–57. doi: 10.1167/iovs.16-19759 27787562 PMC5089211

[17] Vasishta S Poojary G Sharma S Gomes SM Bhat SM Mallya S . Glucose induces DNMT1/IMPDH2-dependent metabolic memory in endothelial cells upon reprograming nucleotide metabolism. FASEB J. (2026) 40:e71374. doi: 10.1096/fj.202502260RR 41445454

[18] Okabe J Orlowski C Balcerczyk A Tikellis C Thomas MC Cooper ME . Distinguishing hyperglycemic changes by Set7 in vascular endothelial cells. Circ Res. (2012) 110:1067–76. doi: 10.1161/CIRCRESAHA.112.266171 22403242

[19] Zhang E Guo Q Gao H Xu R Teng S Wu Y . Metformin and resveratrol inhibited high glucose-induced metabolic memory of endothelial senescence through the SIRT1/p300/p53/p21 pathway. PloS One. (2015) 10:e0143814. doi: 10.1371/journal.pone.0143814 26629991 PMC4668014

[20] Edgar L Akbar N Braithwaite AT Krausgruber T Gallart-Ayala H Bailey J . Hyperglycemia induces trained immunity in macrophages and their precursors and promotes atherosclerosis. Circulation. (2021) 144:961–82. doi: 10.1161/CIRCULATIONAHA.120.046464 34255973 PMC8448412

[21] Yao Y Song Q Hu C Da X Yu Y He Z . Endothelial cell metabolic memory causes cardiovascular dysfunction in diabetes. Cardiovasc Res. (2022) 118:196–211. doi: 10.1093/cvr/cvab013 33483741

[22] Costantino S Paneni F Lüscher TF Cosentino F . MicroRNA profiling unveils hyperglycaemic memory in the diabetic heart. Eur Heart J. (2016) 37:572–6. doi: 10.1093/eurheartj/ehv599 26553540

[23] Zhan J Jin K Ding N Zhou Y Hu G Yuan S . Positive feedback loop of miR-320 and CD36 regulates the hyperglycemic memory-induced diabetic diastolic cardiac dysfunction. Mol Ther Nucleic Acids. (2023) 31:122–38. doi: 10.1016/j.omtn.2022.12.009 36618264 PMC9813582

[24] Shahzad K Gadi I Naïr S Al-Dabet MM Kohli S Bock F . Activated protein C reverses epigenetically sustained p66^Shc^ expression in plaque-associated macrophages in diabetes. Commun Biol. (2018) 1:104. doi: 10.1038/s42003-018-0108-5 30271984 PMC6123684

[25] Al-Dabet MM Shahzad K Elwakiel A Sulaj A Kopf S Bock F . Reversal of the renal hyperglycemic memory in diabetic kidney disease by targeting sustained tubular p21 expression. Nat Commun. (2022) 13:5062. doi: 10.1038/s41467-022-32477-9 36030260 PMC9420151

[26] Zhao J Yang S Shu B Chen L Yang R Xu Y . Transient high glucose causes persistent vascular dysfunction and delayed wound healing by the DNMT1-mediated Ang-1/NF-κB pathway. J Invest Dermatol. (2021) 141:1573–84. doi: 10.1016/j.jid.2020.10.023 33259831

[27] Wilson-Verdugo M Bustos-García B Adame-Guerrero O Hersch-González J Cano-Domínguez N Soto-Nava M . Reversal of high-glucose-induced transcriptional and epigenetic memories through NRF2 pathway activation. Life Sci Alliance. (2024) 7:e202302382. doi: 10.26508/lsa.202302382 38755006 PMC11099870

[28] Zhong Q Kowluru RA . Epigenetic modification of Sod2 in the development of diabetic retinopathy and in the metabolic memory: role of histone methylation. Invest Ophthalmol Vis Sci. (2013) 54:244–50. doi: 10.1167/iovs.12-10854 23221071 PMC3590072

[29] Mohammad G Kowluru RA . Mitochondrial dynamics in the metabolic memory of diabetic retinopathy. J Diabetes Res. (2022) 2022:3555889. doi: 10.1155/2022/3555889 35399705 PMC8989559

[30] Kowluru RA Alka K . Mitochondrial quality control and metabolic memory phenomenon associated with continued progression of diabetic retinopathy. Int J Mol Sci. (2023) 24:8076. doi: 10.3390/ijms24098076 37175784 PMC10179288

[31] Kowluru RA Mohammad G Kumar J . Impaired removal of the damaged mitochondria in the metabolic memory phenomenon associated with continued progression of diabetic retinopathy. Mol Neurobiol. (2024) 61:188–99. doi: 10.1007/s12035-023-03534-1 37596436 PMC10791911

[32] Santos JM Kowluru RA . Impaired transport of mitochondrial transcription factor A and the metabolic memory phenomenon associated with the progression of diabetic retinopathy. Diabetes Metab Res Rev. (2013) 29:204–13. doi: 10.1002/dmrr.2384 23255365 PMC3596457

[33] Santos JM Mishra M Kowluru RA . Posttranslational modification of mitochondrial transcription factor A in impaired mitochondria biogenesis: implications in diabetic retinopathy and metabolic memory phenomenon. Exp Eye Res. (2014) 121:168–77. doi: 10.1016/j.exer.2014.02.010 24607487 PMC4036231

[34] Paneni F Mocharla P Akhmedov A Costantino S Osto E Volpe M . Gene silencing of the mitochondrial adaptor p66^Shc^ suppresses vascular hyperglycemic memory in diabetes. Circ Res. (2012) 111:278–89. doi: 10.1161/CIRCRESAHA.112.266593 22693349

[35] Wellen KE Hatzivassiliou G Sachdeva UM Bui TV Cross JR Thompson CB . ATP-citrate lyase links cellular metabolism to histone acetylation. Science. (2009) 324:1076–80. doi: 10.1126/science.1164097 19461003 PMC2746744

[36] Mentch SJ Mehrmohamadi M Huang L Liu X Gupta D Mattocks D . Histone methylation dynamics and gene regulation occur through the sensing of one-carbon metabolism. Cell Metab. (2015) 22:861–73. doi: 10.1016/j.cmet.2015.08.024 26411344 PMC4635069

[37] Baksh SC Finley LWS . Metabolic coordination of cell fate by α-ketoglutarate-dependent dioxygenases. Trends Cell Biol. (2021) 31:24–36. doi: 10.1016/j.tcb.2020.09.010 33092942 PMC7748998

[38] de Oliveira AA de Oliveira TF Bobadilla LL Garcia CC Berra CM de Souza-Pinto NC . Sustained kidney biochemical derangement in treated experimental diabetes: a clue to metabolic memory. Sci Rep. (2017) 7:40544. doi: 10.1038/srep40544 28079150 PMC5228190

[39] Cheng SC Quintin J Cramer RA Shepardson KM Saeed S Kumar V . mTOR- and HIF-1α-mediated aerobic glycolysis as metabolic basis for trained immunity. Science. (2014) 345:1250684. doi: 10.1126/science.1250684 25258083 PMC4226238

[40] Bekkering S Arts RJW Novakovic B Kourtzelis I van der Heijden CDCC Li Y . Metabolic induction of trained immunity through the mevalonate pathway. Cell. (2018) 172:135–146.e9. doi: 10.1016/j.cell.2017.11.025 29328908

[41] Keating ST Groh L Thiem K Bekkering S Li Y Matzaraki V . Rewiring of glucose metabolism defines trained immunity induced by oxidized low-density lipoprotein. J Mol Med (Berl). (2020) 98:819–31. doi: 10.1007/s00109-020-01915-w 32350546 PMC7297856

[42] Ihnat MA Thorpe JE Kamat CD Szabó C Green DE Warnke LA . Reactive oxygen species mediate a cellular ‘memory’ of high glucose stress signalling. Diabetologia. (2007) 50:1523–31. doi: 10.1007/s00125-007-0684-2 17508197

[43] Ding M Shi R Du Y Chang P Gao T De D . O-GlcNAcylation-mediated endothelial metabolic memory contributes to cardiac damage via small extracellular vesicles. Cell Metab. (2025) 37:1344–1363.e6. doi: 10.1016/j.cmet.2025.03.006 40179889

[44] Bierhaus A Schiekofer S Schwaninger M Andrassy M Humpert PM Chen J . Diabetes-associated sustained activation of the transcription factor nuclear factor-κB. Diabetes. (2001) 50:2792–808. doi: 10.2337/diabetes.50.12.2792 11723063

[45] Arden C Park SH Yasasilka XR Lee EY Lee MS . Autophagy and lysosomal dysfunction in diabetes and its complications. Trends Endocrinol Metab. (2024) 35:1078–90. doi: 10.1016/j.tem.2024.06.010 39054224

[46] Kumar S Pamulapati H Tikoo K . Fatty acid induced metabolic memory involves alterations in renal histone H3K36me2 and H3K27me3. Mol Cell Endocrinol. (2016) 422:233–42. doi: 10.1016/j.mce.2015.12.019 26747726

[47] Siersbæk M Varticovski L Yang S Baek S Nielsen R Mandrup S . High fat diet-induced changes of mouse hepatic transcription and enhancer activity can be reversed by subsequent weight loss. Sci Rep. (2017) 7:40220. doi: 10.1038/srep40220 28071704 PMC5223143

[48] Du XL Edelstein D Dimmeler S Ju Q Sui C Brownlee M . Hyperglycemia inhibits endothelial nitric oxide synthase activity by posttranslational modification at the Akt site. J Clin Invest. (2001) 108:1341–8. doi: 10.1172/JCI11235 11696579 PMC209429

[49] Haidari M Zhang W Willerson JT Dixon RAF . Disruption of endothelial adherens junctions by high glucose is mediated by protein kinase C-β-dependent vascular endothelial cadherin tyrosine phosphorylation. Cardiovasc Diabetol. (2014) 13:112. doi: 10.1186/1475-2840-13-112 25927959 PMC4223716

[50] Sawada N Jiang A Takizawa F Safdar A Manika A Tesmenitsky Y . Endothelial PGC-1α mediates vascular dysfunction in diabetes. Cell Metab. (2014) 19:246–58. doi: 10.1016/j.cmet.2013.12.014 24506866 PMC4040246

[51] Mohammed SA Gorica E Albiero M Karsai G Mengozzi A Caravaggi CM . Targeting SETD7 rescues diabetes-induced impairment of angiogenic response by transcriptional repression of semaphorin-3G. Diabetes. (2025) 74:969–82. doi: 10.2337/db24-0997 40014026

[52] Lu L Zhu M Wu Q Sun Z Chen X Miao C . Sirt7/HIC1 complex participates in hyperglycaemia-mediated EndMT via modulation of SDC1 expression in diabetic kidney disease and metabolic memory. J Cell Mol Med. (2024) 28:e18336. doi: 10.1111/jcmm.18336 38686489 PMC11058670

[53] Yan J Tie G Wang S Tutto A DeMarco N Khair L . Diabetes impairs wound healing by DNMT1-dependent dysregulation of hematopoietic stem-cell differentiation towards macrophages. Nat Commun. (2018) 9:33. doi: 10.1038/s41467-017-02425-z 29295997 PMC5750226

[54] Thiem K Keating ST Netea MG Riksen NP Tack CJ van Diepen J . Hyperglycemic memory of innate immune cells promotes *in vitro* proinflammatory responses of human monocytes and murine macrophages. J Immunol. (2021) 206:807–13. doi: 10.4049/jimmunol.1901348 33431659

[55] Flynn MC Kraakman MJ Tikellis C Lee MKS Hanssen NMJ Kammoun HL . Transient intermittent hyperglycemia accelerates atherosclerosis by promoting myelopoiesis. Circ Res. (2020) 127:877–92. doi: 10.1161/CIRCRESAHA.120.316653 32564710 PMC7486277

[56] Maione AG Brudno Y Stojadinovic O Park LK Smith A Tellechea A . Three-dimensional human tissue models that incorporate diabetic foot ulcer-derived fibroblasts mimic *in vivo* features of chronic wounds. Tissue Eng Part C Methods. (2015) 21:499–508. doi: 10.1089/ten.TEC.2014.0414 25343343 PMC4410281

[57] Pastar I Marjanovic J Liang L Stone RC Kashpur O Jozic I . Cellular reprogramming of diabetic foot ulcer fibroblasts triggers a pro-healing miRNA-mediated epigenetic signature. Exp Dermatol. (2021) 30:1065–72. doi: 10.1111/exd.14405 34114688

[58] Mohanty SK Singh K Kumar M Verma SS Srivastava R Gnyawali SC . Vasculogenic skin reprogramming requires TET-mediated gene demethylation in fibroblasts for rescuing impaired perfusion in diabetes. Nat Commun. (2024) 15:10277. doi: 10.1038/s41467-024-54385-w 39604331 PMC11603198

[59] Liu F Lagares D Choi KM Stopfer L Marinković A Vrbanac V . Mechanosignaling through YAP and TAZ drives fibroblast activation and fibrosis. Am J Physiol Lung Cell Mol Physiol. (2015) 308:L344–57. doi: 10.1152/ajplung.00300.2014 25502501 PMC4329470

[60] Bauer TM Mangum KD Buckley SD Shadiow J Joshi AD Audu CO . TNF-α represses fibroblast-to-myofibroblast transition through the histone methyltransferase SETDB2. JCI Insight. (2025) 10:e190836. doi: 10.1172/jci.insight.190836 41277557 PMC12643505

[61] Guo S Dai C Guo M Taylor B Harmon JS Sander M . Inactivation of specific β cell transcription factors in type 2 diabetes. J Clin Invest. (2013) 123:3305–16. doi: 10.1172/JCI65390 23863625 PMC3726150

[62] Talchai C Xuan S Lin HV Sussel L Accili D . Pancreatic β cell dedifferentiation as a mechanism of diabetic β cell failure. Cell. (2012) 150:1223–34. doi: 10.1016/j.cell.2012.07.029 22980982 PMC3445031

[63] Gao T McKenna B Li C Reichert M Nguyen J Singh T . Pdx1 maintains β cell identity and function by repressing an α cell program. Cell Metab. (2014) 19:259–71. doi: 10.1016/j.cmet.2013.12.002 24506867 PMC3950964

[64] Taylor BL Liu FF Sander M . Nkx6.1 is essential for maintaining the functional state of pancreatic beta cells. Cell Rep. (2013) 4:1262–75. doi: 10.1016/j.celrep.2013.08.010 24035389 PMC4058003

[65] Butler AE Robertson RP Hernandez R Matveyenko AV Gurlo T Butler PC . Beta cell nuclear musculoaponeurotic fibrosarcoma oncogene family A (MafA) is deficient in type 2 diabetes. Diabetologia. (2012) 55:2985–8. doi: 10.1007/s00125-012-2666-2 22847061 PMC3556170

[66] Gauthier BR Wiederkehr A Baquié M Dai C Powers AC Kerr-Conte J . PDX1 deficiency causes mitochondrial dysfunction and defective insulin secretion through TFAM suppression. Cell Metab. (2009) 10:110–8. doi: 10.1016/j.cmet.2009.07.002 19656489 PMC4012862

[67] Cinti F Bouchi R Kim-Muller JY Ohmura Y Sandoval PR Masini M . Evidence of β-cell dedifferentiation in human type 2 diabetes. J Clin Endocrinol Metab. (2016) 101:1044–54. doi: 10.1210/jc.2015-2860 26713822 PMC4803182

[68] Chen CW Guan BJ Alzahrani MR Gao Z Gao L Bracey S . Adaptation to chronic ER stress enforces pancreatic β-cell plasticity. Nat Commun. (2022) 13:4621. doi: 10.1038/s41467-022-32425-7 35941159 PMC9360004

[69] Wang Z York NW Nichols CG Remedi MS . Pancreatic β cell dedifferentiation in diabetes and redifferentiation following insulin therapy. Cell Metab. (2014) 19:872–82. doi: 10.1016/j.cmet.2014.03.010 24746806 PMC4067979

[70] Furth-Lavi J Hija A Tornovsky-Babeay S Mazouz A Dahan T Stolovich-Rain M . Glycemic control releases regenerative potential of pancreatic beta cells blocked by severe hyperglycemia. Cell Rep. (2022) 41:111719. doi: 10.1016/j.celrep.2022.111719 36450253 PMC9789023

[71] Aguayo-Mazzucato C Andle J Lee TB Jr Midha A Talemal L Chipashvili V . Acceleration of β cell aging determines diabetes and senolysis improves disease outcomes. Cell Metab. (2019) 30:129–142.e4. doi: 10.1016/j.cmet.2019.05.006 31155496 PMC6610720

[72] Usui ML Mansbridge JN Carter WG Fujita M Olerud JE . Keratinocyte migration, proliferation, and differentiation in chronic ulcers from patients with diabetes and normal wounds. J Histochem Cytochem. (2008) 56:687–96. doi: 10.1369/jhc.2008.951194 18413645 PMC2430161

[73] Lan CCE Liu IH Fang AH Wen CH Wu CS . Hyperglycaemic conditions decrease cultured keratinocyte mobility: implications for impaired wound healing in patients with diabetes. Br J Dermatol. (2008) 159:1103–15. doi: 10.1111/j.1365-2133.2008.08789.x 18717678

[74] Li L Zhang J Zhang Q Zhang D Xiang F Jia J . High glucose suppresses keratinocyte migration through the inhibition of p38 MAPK/autophagy pathway. Front Physiol. (2019) 10:24. doi: 10.3389/fphys.2019.00024 30745880 PMC6360165

[75] Zhang C Ponugoti B Tian C Xu F Tarapore R Batres A . FOXO1 differentially regulates both normal and diabetic wound healing. J Cell Biol. (2015) 209:289–303. doi: 10.1083/jcb.201409032 25918228 PMC4411275

[76] Lan CCE Wu CS Huang SM Wu IH Chen GS . High-glucose environment enhanced oxidative stress and increased interleukin-8 secretion from keratinocytes: new insights into impaired diabetic wound healing. Diabetes. (2013) 62:2530–8. doi: 10.2337/db12-1714 23423570 PMC3712048

[77] Huang SM Wu CS Chiu MH Wu CH Chang YT Chen GS . High glucose environment induces M1 macrophage polarization that impairs keratinocyte migration via TNF-α: an important mechanism to delay diabetic wound healing. J Dermatol Sci. (2019) 96:159–67. doi: 10.1016/j.jdermsci.2019.11.004 31761388

[78] Rizwan H Kumar S Kumari G Pal A . High glucose-induced increasing reactive nitrogen species accumulation triggered mitochondrial dysfunction, inflammation, and apoptosis in keratinocytes. Life Sci. (2023) 312:121208. doi: 10.1016/j.lfs.2022.121208 36427546

[79] Shu F Gao H Wu W Yu S Zhang L Liu H . Amniotic epithelial cells accelerate diabetic wound healing by protecting keratinocytes and fibroblasts from high-glucose-induced senescence. Cell Biol Int. (2022) 46:755–70. doi: 10.1002/cbin.11771 35077602

[80] Liu Y Wang P Li J Chen L Shu B Wang H . Single-cell RNA sequencing reveals the impaired epidermal differentiation and pathological microenvironment in diabetic foot ulcer. Burns Trauma. (2025) 13:tkae065. doi: 10.1093/burnst/tkae065 40040959 PMC11879498

[81] Theocharidis G Thomas BE Sarkar D Mumme HL Pilcher WJR Dwivedi B . Single-cell transcriptomic landscape of diabetic foot ulcers. Nat Commun. (2022) 13:181. doi: 10.1038/s41467-021-27801-8 35013299 PMC8748704

[82] Wilkinson HN Clowes C Banyard KL Matteuci P Mace KA Hardman MJ . Elevated local senescence in diabetic wound healing is linked to pathological repair via CXCR2. J Invest Dermatol. (2019) 139:1171–1181.e6. doi: 10.1016/j.jid.2019.01.005 30684552

[83] Lizotte F Denhez B Guay A Gévry N Côté AM Geraldes P . Persistent insulin resistance in podocytes caused by epigenetic changes of SHP-1 in diabetes. Diabetes. (2016) 65:3705–17. doi: 10.2337/db16-0254 27585521

[84] Li X Liu J Lu L Huang T Hou W Wang F . Sirt7 associates with ELK1 to participate in hyperglycemia memory and diabetic nephropathy via modulation of DAPK3 expression and endothelial inflammation. Transl Res. (2022) 247:99–116. doi: 10.1016/j.trsl.2022.04.005 35470010

[85] Herman-Edelstein M Scherzer P Tobar A Levi M Gafter U . Altered renal lipid metabolism and renal lipid accumulation in human diabetic nephropathy. J Lipid Res. (2014) 55:561–72. doi: 10.1194/jlr.P040501 24371263 PMC3934740

[86] Abedini A Levinsohn J Klötzer KA Dumoulin B Ma Z Frederick J . Single-cell multi-omic and spatial profiling of human kidneys implicates the fibrotic microenvironment in kidney disease progression. Nat Genet. (2024) 56:1712–24. doi: 10.1038/s41588-024-01802-x 39048792 PMC11592391

[87] Tanwar VS Malek V Wang J Luo Y Malhi NK Zhang H . Single-cell multimodal profiling highlights persistent aortic smooth muscle cell changes in diabetic mice despite glycemic control. Arterioscler Thromb Vasc Biol. (2026) 46:e324012. doi: 10.1161/ATVBAHA.125.324012 41641545 PMC12916121

[88] Friedrichs P Schlotterer A Sticht C Kolibabka M Wohlfart P Dietrich A . Hyperglycaemic memory affects the neurovascular unit of the retina in a diabetic mouse model. Diabetologia. (2017) 60:1354–8. doi: 10.1007/s00125-017-4254-y 28321468

[89] Hammes HP Lin J Wagner P Feng Y Vom Hagen F Krzizok T . Angiopoietin-2 causes pericyte dropout in the normal retina: evidence for involvement in diabetic retinopathy. Diabetes. (2004) 53:1104–10. doi: 10.2337/diabetes.53.4.1104 15047628

[90] Kowluru RA Zhong Q Kanwar M . Metabolic memory and diabetic retinopathy: role of inflammatory mediators in retinal pericytes. Exp Eye Res. (2010) 90:617–23. doi: 10.1016/j.exer.2010.02.006 20170650 PMC2857535

[91] Rungger-Brändle E Dosso AA Leuenberger PM . Glial reactivity, an early feature of diabetic retinopathy. Invest Ophthalmol Vis Sci. (2000) 41:1971–80. 10845624

[92] Masser DR VanGuilder Starkey HD Bixler GV Dunton W Bronson SK Freeman WM . Insulin treatment normalizes retinal neuroinflammation but not markers of synapse loss in diabetic rats. Exp Eye Res. (2014) 125:95–106. doi: 10.1016/j.exer.2014.06.005 24931083 PMC4122586

[93] Loesche M Gardner SE Kalan L Horwinski J Zheng Q Hodkinson BP . Temporal stability in chronic wound microbiota is associated with poor healing. J Invest Dermatol. (2017) 137:237–44. doi: 10.1016/j.jid.2016.08.009 27566400 PMC5279919

[94] Kalan LR Meisel JS Loesche MA Horwinski J Soaita I Chen X . Strain- and species-level variation in the microbiome of diabetic wounds is associated with clinical outcomes and therapeutic efficacy. Cell Host Microbe. (2019) 25:641–655.e5. doi: 10.1016/j.chom.2019.03.006 31006638 PMC6526540

[95] Sen CK Gordillo GM Roy S Jahnke J Sinha M Timsina L . High transepidermal water loss at the site of wound closure is associated with increased recurrence of diabetic foot ulcers. Diabetes Care. (2025) 48:1233–40. doi: 10.2337/dc25-0300 40445117 PMC12178626

[96] Bus SA Waaijman R Arts M de Haart M Busch-Westbroek T van Baal J . Effect of custom-made footwear on foot ulcer recurrence in diabetes: a multicenter randomized controlled trial. Diabetes Care. (2013) 36:4109–16. doi: 10.2337/dc13-0996 24130357 PMC3836114

[97] Bus SA aan de Stegge WB van Baal JG Busch-Westbroek TE Nollet F van Netten JJ . Effectiveness of at-home skin temperature monitoring in reducing the incidence of foot ulcer recurrence in people with diabetes: a multicenter randomized controlled trial (DIATEMP). BMJ Open Diabetes Res Care. (2021) 9:e002392. doi: 10.1136/bmjdrc-2021-002392 34493496 PMC8424833

[98] Loughlin DT Artlett CM . 3-Deoxyglucosone-collagen alters human dermal fibroblast migration and adhesion: implications for impaired wound healing in patients with diabetes. Wound Repair Regener. (2009) 17:739–49. doi: 10.1111/j.1524-475X.2009.00532.x 19769726

[99] Thaiss CA Levy M Grosheva I Zheng D Soffer E Blacher E . Hyperglycemia drives intestinal barrier dysfunction and risk for enteric infection. Science. (2018) 359:1376–83. doi: 10.1126/science.aar3318 29519916

[100] Christ A Günther P Lauterbach MAR Duewell P Biswas D Pelka K . Western diet triggers NLRP3-dependent innate immune reprogramming. Cell. (2018) 172:162–175.e14. doi: 10.1016/j.cell.2017.12.013 29328911 PMC6324559

[101] Koh A Molinaro A Ståhlman M Khan MT Schmidt C Mannerås-Holm L . Microbially produced imidazole propionate impairs insulin signaling through mTORC1. Cell. (2018) 175:947–961.e17. doi: 10.1016/j.cell.2018.09.055 30401435

[102] Gæde P Oellgaard J Carstensen B Rossing P Lund-Andersen H Parving HH . Years of life gained by multifactorial intervention in patients with type 2 diabetes mellitus and microalbuminuria: 21 years follow-up on the Steno-2 randomised trial. Diabetologia. (2016) 59:2298–307. doi: 10.1007/s00125-016-4065-6 27531506 PMC5506099

[103] Ceriello A Esposito K Piconi L Ihnat MA Thorpe JE Testa R . Oscillating glucose is more deleterious to endothelial function and oxidative stress than mean glucose in normal and type 2 diabetic patients. Diabetes. (2008) 57:1349–54. doi: 10.2337/db08-0063 18299315

[104] Poulaki V Qin W Joussen AM Hurlbut P Wiegand SJ Rudge J . Acute intensive insulin therapy exacerbates diabetic blood-retinal barrier breakdown via hypoxia-inducible factor-1α and VEGF. J Clin Invest. (2002) 109:805–15. doi: 10.1172/JCI13776 11901189 PMC150907

[105] Chandrasekaran K Najimi N Sagi AR Yarlagadda S Salimian M Arvas MI . NAD^+^ precursors repair mitochondrial function in diabetes and prevent experimental diabetic neuropathy. Int J Mol Sci. (2022) 23:4887. doi: 10.3390/ijms23094887 35563288 PMC9102948

[106] Kim MN Moon JH Cho YM . Sodium–glucose cotransporter-2 inhibition reduces cellular senescence in the diabetic kidney by promoting ketone body-induced NRF2 activation. Diabetes Obes Metab. (2021) 23:2561–71. doi: 10.1111/dom.14503 34318973

[107] Bekkering S Stiekema LCA Bernelot Moens S Verweij SL Novakovic B Prange K . Treatment with statins does not revert trained immunity in patients with familial hypercholesterolemia. Cell Metab. (2019) 30:1–2. doi: 10.1016/j.cmet.2019.05.014 31204280

[108] Crespo-Garcia S Fournier F Diaz-Marin R Klier S Ragusa D Masaki L . Therapeutic targeting of cellular senescence in diabetic macular edema: preclinical and phase 1 trial results. Nat Med. (2024) 30:443–54. doi: 10.1038/s41591-024-02802-4 38321220

[109] Klier S Dananberg J Masaki L Bhisitkul RB Khanani AM Maturi R . Safety and efficacy of senolytic UBX1325 in diabetic macular edema. NEJM Evid. (2025) 4:EVIDoa2400009. doi: 10.1056/EVIDoa2400009 40261111

[110] Guo X Wen S Wang J Zeng X Yu H Chen Y . Senolytic combination of dasatinib and quercetin attenuates renal damage in diabetic kidney disease. Phytomedicine. (2024) 130:155705. doi: 10.1016/j.phymed.2024.155705 38761776

[111] Hickson LJ Langhi Prata LGP Bobart SA Evans TK Giorgadze N Hashmi SK . Senolytics decrease senescent cells in humans: preliminary report from a clinical trial of dasatinib plus quercetin in individuals with diabetic kidney disease. EBioMedicine. (2019) 47:446–56. doi: 10.1016/j.ebiom.2019.08.069 31542391 PMC6796530

[112] Samarawickrama PN Zhang G Zhu E Dong X Nisar A Zhu H . Clearance of senescent cells enhances skin wound healing in type 2 diabetic mice. Theranostics. (2024) 14:5429–42. doi: 10.7150/thno.100991 39310100 PMC11413796

[113] Pan R Ren J Chen X Flores LF Gonzalez RVL Adonnino AA . Editing DNA methylation *in vivo*. Nat Commun. (2026) 17:527. doi: 10.1038/s41467-025-67222-5 41372159 PMC12804732

[114] Zinman B Lachin JM Inzucchi SE . Empagliflozin, cardiovascular outcomes, and mortality in type 2 diabetes. N Engl J Med. (2015) 373:2117–28. doi: 10.1056/NEJMoa1504720 26378978

[115] Sylvester RD Khong TK . Dapagliflozin in patients with chronic kidney disease. N Engl J Med. (2020) 383:1436–46. doi: 10.1056/NEJMoa2024816 32970396

[116] Perkovic V Tuttle KR Rossing P Mahaffey KW Mann JFE Bakris G . Effects of semaglutide on chronic kidney disease in patients with type 2 diabetes. N Engl J Med. (2024) 391:109–21. doi: 10.1056/NEJMoa2403347 38785209

[117] Bjornstad P Choi YJ Narongkiatikhun P Karihaloo A Prasad PV Li LP . SGLT2 inhibition modulates metabolic, vascular, and inflammatory molecular markers in the kidney in youth with type 1 diabetes. Sci Transl Med. (2026) 18:eaee1005. doi: 10.1126/scitranslmed.aee1005 42485434

[118] Scisciola L Rizzo MR Cataldo V Fontanella RA Balestrieri ML D'Onofrio N . Incretin drugs effect on epigenetic machinery: new potential therapeutic implications in preventing vascular diabetic complications. FASEB J. (2020) 34:16489–503. doi: 10.1096/fj.202000860RR 33090591

[119] Pfützner A Weise A Pfützner-Riehn E Lübben G Morcos M Karagiannis E . Downregulation of the proinflammatory state of circulating mononuclear cells by short-term treatment with pioglitazone in patients with type 2 diabetes mellitus and coronary artery disease. PPAR Res. (2011) 2011:647017. doi: 10.1155/2011/647017 21808640 PMC3144663

[120] Ma S Zhang B LaFave LM Earl AS Chiang Z Hu Y . Chromatin potential identified by shared single-cell profiling of RNA and chromatin. Cell. (2020) 183:1103–1116.e20. doi: 10.1016/j.cell.2020.09.056 33098772 PMC7669735

[121] Hu Y Huang K An Q Du G Hu G Xue J . Simultaneous profiling of transcriptome and DNA methylome from a single cell. Genome Biol. (2016) 17:88. doi: 10.1186/s13059-016-0950-z 27150361 PMC4858893

[122] Wang Z Fu W Huo M He B Liu Y Tian L . Spatial-resolved metabolomics reveals tissue-specific metabolic reprogramming in diabetic nephropathy by using mass spectrometry imaging. Acta Pharm Sin B. (2021) 11:3665–77. doi: 10.1016/j.apsb.2021.05.013 34900545 PMC8642449

[123] Liu Z Gu Y Chakarov S Bleriot C Kwok I Chen X . Fate mapping via Ms4a3-expression history traces monocyte-derived cells. Cell. (2019) 178:1509–1525.e19. doi: 10.1016/j.cell.2019.08.009 31491389

[124] Biddy BA Kong W Kamimoto K Guo C Waye SE Sun T . Single-cell mapping of lineage and identity in direct reprogramming. Nature. (2018) 564:219–24. doi: 10.1038/s41586-018-0744-4 30518857 PMC6635140

[125] Bowling S Sritharan D Osorio FG Nguyen M Cheung P Rodriguez-Fraticelli A . An engineered CRISPR–Cas9 mouse line for simultaneous readout of lineage histories and gene expression profiles in single cells. Cell. (2020) 181:1410–1422.e27. doi: 10.1016/j.cell.2020.04.048 32413320 PMC7529102

[126] Dixit A Parnas O Li B Chen J Fulco CP Jerby-Arnon L . Perturb-Seq: dissecting molecular circuits with scalable single-cell RNA profiling of pooled genetic screens. Cell. (2016) 167:1853–1866.e17. doi: 10.1016/j.cell.2016.11.038 27984732 PMC5181115

[127] Metzner E Southard KM Norman TM . Multiome Perturb-seq unlocks scalable discovery of integrated perturbation effects on the transcriptome and epigenome. Cell Syst. (2025) 16:101161. doi: 10.1016/j.cels.2024.12.002 39689711 PMC11738662

[128] de Lemos L Antas P Ferreira IS Santos IP Felgueiras B Gomes CM . Modelling neurodegeneration and inflammation in early diabetic retinopathy using 3D human retinal organoids. In Vitro Models. (2024) 3:33–48. doi: 10.1007/s44164-024-00068-1 39872068 PMC11756505

[129] Wang L Tao T Su W Yu H Yu Y Qin J . A disease model of diabetic nephropathy in a glomerulus-on-a-chip microdevice. Lab Chip. (2017) 17:1749–60. doi: 10.1039/C7LC00134G 28418422

[130] García-Calzón S Maguolo A Eichelmann F Edsfeldt A Perfilyev A Maziarz M . Epigenetic biomarkers predict macrovascular events in individuals with type 2 diabetes. Cell Rep Med. (2025) 6:102290. doi: 10.1016/j.xcrm.2025.102290 40780200 PMC12432358

[131] Sharma K Zhang G Hansen J Bjornstad P Lee HJ Menon R . Endogenous adenine mediates kidney injury in diabetic models and predicts diabetic kidney disease in patients. J Clin Invest. (2023) 133:e170341. doi: 10.1172/JCI170341 37616058 PMC10575723

[132] Gerrits EG Lutgers HL Kleefstra N Graaff R Groenier KH Smit AJ . Skin autofluorescence: a tool to identify type 2 diabetic patients at risk for developing microvascular complications. Diabetes Care. (2008) 31:517–21. doi: 10.2337/dc07-1755 18039805

[133] Sun Z Tang F Wong R Lok J Szeto SKH Chan JCK . OCT angiography metrics predict progression of diabetic retinopathy and development of diabetic macular edema: a prospective study. Ophthalmology. (2019) 126:1675–84. doi: 10.1016/j.ophtha.2019.06.016 31358386

[134] Chen Z Miao F Braffett BH Lachin JM Zhang L Wu X . DNA methylation mediates development of HbA1c-associated complications in type 1 diabetes. Nat Metab. (2020) 2:744–62. doi: 10.1038/s42255-020-0231-8 32694834 PMC7590966

[135] Genuth S Sun W Cleary P Sell DR Dahms W Malone J . Glycation and carboxymethyllysine levels in skin collagen predict the risk of future 10-year progression of diabetic retinopathy and nephropathy in the diabetes control and complications trial and epidemiology of diabetes interventions and complications participants with type 1 diabetes. Diabetes. (2005) 54:3103–11. doi: 10.2337/diabetes.54.11.3103 16249432 PMC2622724

[136] Kowluru RA Mohammad G . Epigenetics and mitochondrial stability in the metabolic memory phenomenon associated with continued progression of diabetic retinopathy. Sci Rep. (2020) 10:6655. doi: 10.1038/s41598-020-63527-1 32313015 PMC7171070

