# Supplementary material for: Metabolic memory in diabetic complications: evidence for persistent cellular states and limits of therapeutic reversal
Source: Front Endocrinol (Lausanne). 2026 Oct 5;17:1954392. doi: 10.3389/fendo.2026.1954392 (PMC13678445; doi:10.3389/fendo.2026.1954392)
Supplement: Supplementary file 1 [file SupplementaryFile1.docx]

# Appendix A Literature selection and revision evidence audit

## A1 Scope and provenance

The review concerns persistent cellular or tissue dysfunction relevant to diabetic kidney disease, cardiovascular complications, retinopathy and diabetic foot disease. β-cell plasticity and non-glucose exposures are comparative models. The original manuscript reported searches of PubMed and Google Scholar through July 2026, but original database-specific strings, retrieval exports, screening counts and selection logs were not retained in the records available for this revision. They cannot be reconstructed reliably. No retrospective count, independent dual-screening process or complete historical query is asserted here.

## A2 Documented supplementary PubMed retrieval

Database and interface: PubMed via NCBI E-utilities (ESearch and EFetch). Search executed 8 September 2026. Publication-date range: 1 January 1900 to 8 September 2026. No species or language filter was applied to retrieval. Review and Editorial publication types were excluded by the query, although indexing can leave narrative material in the result set. Retrieval returned 108 unique PubMed identifiers; all 108 titles were inspected. Abstract records were retrieved for the set. The focused query is not designed to retrieve every clinical legacy, trained-immunity or structural-injury study.

("metabolic memory"[Title/Abstract] OR "hyperglycemic memory"[Title/Abstract] OR "hyperglycaemic memory"[Title/Abstract]) AND (diabet*[Title/Abstract] OR glucose[Title/Abstract]) AND (washout[Title/Abstract] OR withdrawal[Title/Abstract] OR normaliz*[Title/Abstract] OR normoglyc*[Title/Abstract] OR revers*[Title/Abstract]) NOT (Review[Publication Type] OR Editorial[Publication Type]) AND ("1900/01/01"[Date - Publication] : "2026/09/08"[Date - Publication])

Google Scholar was not rerun for this supplementary audit. Exact-title and DOI source checks used PubMed, PubMed Central and publisher pages for selected original references. These citation checks are separate from the 108-record PubMed result set and are not represented as a second systematic database search. The earlier exploratory broad memory-term query was not used as the screening set.

## A3 Eligibility and selection rules

For direct cellular-memory evidence, priority was given to original studies defining an initiating metabolic exposure and a subsequent recovery or transfer phase, with measured persistent molecular features linked to function or a standardized challenge response. For causal maintenance, intervention timing had to be identifiable relative to withdrawal; encoding-only interventions were not classified as maintenance rescue. For sustained molecular and functional recovery after treatment cessation, assessment had to extend beyond intervention clearance and include both the underlying state and function. A positive finding was not an eligibility requirement.

Clinical trials were retained to establish legacy effects; patient tissues or cross-sectional omics were retained for clinical relevance and explicitly labelled associative when withdrawal or causality was not tested. Studies of continuing hyperglycaemia, hypoxia, infection or other active stress, basic metabolic–chromatin biology and technology platforms were used as context, not direct proof of cellular memory. Non-original reports, unrelated diseases, unverified claims, duplicate versions and out-of-scope organ systems were not prioritized for the focused mechanistic audit. Additional relevant reports not prioritized are not thereby judged negative or invalid.

Title assessment prioritized 23 reports for closer abstract/source appraisal because they directly tested the reviewer-contested mechanisms, clarified recovery-phase intervention timing, or supplied limiting or contrary evidence. The other 85 retrieved records were not taken forward into this focused audit. This was a purposive narrative selection, not exhaustive full-text eligibility screening. No dual independent screening, formal risk-of-bias instrument, meta-analysis or study-level prevalence estimate was performed. The 23 selections are listed below with the reason for prioritization; landmark clinical and complementary original references were evaluated separately by citation.

## A4 Evidence extraction and conflicting results

The appraisal recorded human/animal/cell origin; exposure and withdrawal design; recovery duration when verified; molecular versus direct activity measurements; functional endpoints; intervention timing; and evidence after intervention withdrawal. Tables 1–3 report these dimensions for representative mechanisms. Abstract-only information was not used to invent washout durations or unreported durability. Missing withdrawal assessment was treated as not demonstrated, rather than as evidence that resetting is impossible.

Recovery and negative results were retained at the claim level. Retinal methylation and pericyte-inflammatory endpoints can improve with longer recovery (16,90); delayed insulin can normalize inflammation while synaptic abnormalities persist (92); most hepatic transcriptional and enhancer changes reversed after weight loss in a comparative diet model (47). Different tissues, exposure durations and endpoints were not pooled as a single contradiction or consensus. Clinical association was not upgraded by proximity to a mechanistic animal study. Narrative selection, incomplete retrieval of poorly indexed mechanisms, language/access constraints and heterogeneous recovery designs remain limitations.

## A5 Prioritized records and reasons for selection

PMID 41445454; reference (17). DNMT1/IMPDH2 coupling; distinguish excess sprouting and clinical association.

PMID 40179889; reference (43). Direct activity and vesicle transfer; distinguish source memory from recipient stimulation.

PMID 38686489; reference (52). EndMT persistence and Sirt7/HIC1 intervention in a defined model.

PMID 37596436; reference (31). Persistent mitophagy-flux defect; organelle function beyond static marks.

PMID 37175784; reference (30). Recovery-phase mitochondrial fusion intervention and respiration endpoints.

PMID 36618264; reference (23). Specific cardiac miR-320/CD36 feedback rather than a universal network.

PMID 36030260; reference (25). Tubular p21/DNMT1 maintenance; separate human observations from animal rescue.

PMID 35470010; reference (84). Sirt7/ELK1/DAPK3 signalling and recovery-phase inflammation.

PMID 30271984; reference (24). Macrophage epigenetic/redox maintenance and glucose-versus-lipid specificity.

PMID 27787562; reference (16). DNMT activity and recovery-phase intervention; improvement with longer recovery.

PMID 26553540; reference (22). Persistent miRNA abundance; avoid equating inferred pathways with direct activity.

PMID 25437049; reference (13). Patient-derived fibroblast methylation persistence; causal limits.

PMID 24931083; reference (92). Inflammatory normalization despite retained synapse-loss markers.

PMID 24607487; reference (33). TFAM post-translational persistence; separate prevention from maintenance.

PMID 23255365; reference (32). TFAM transport/binding during prolonged animal recovery.

PMID 23221071; reference (28). SOD2 chromatin persistence; distinguish encoding intervention from delayed rescue.

PMID 22693349; reference (34). Direct p66Shc activity, function and normalization-phase silencing.

PMID 20170650; reference (90). Retinal pericyte inflammatory persistence depends on exposure/recovery duration.

PMID 38755006; reference (27). New citation: NRF2-mediated molecular state modification after induction.

PMID 33259831; reference (26). New citation: DNMT1/Ang-1 links epigenetic regulation to persistent vascular dysfunction.

PMID 28071704; reference (47). New citation: comparative hepatic recovery limits universal irreversibility claims.

PMID 26747726; reference (46). New citation: lipid-withdrawal signalling phenotype with associative histone changes.

PMID 41641545; reference (87). New citation: recent aortic persistence with partial recovery and indirect pathway inference.

## A6 Complete PubMed retrieval identifiers

The following 108 identifiers identify the actual retrieval set, not 108 included mechanistic studies. NCBI indexing changes can alter a later rerun.

42669882; 42522575; 42010679; 41951709; 41744800; 41641545; 41445454; 41227374; 41088293; 40661578; 40574282; 40488531; 40179889; 40019194; 39558680; 39539089; 39525640; 39026923.

38755006; 38749010; 38686489; 37596436; 37405607; 37206566; 37175784; 37152887; 37066707; 36782199; 36618264; 36331561; 36286816; 36212957; 36030260; 35470010; 34944423; 34561995.

34447105; 34352784; 33887258; 33472942; 33259831; 33251641; 33200530; 33149857; 32582175; 32313015; 31902112; 31894851; 31638409; 31462079; 31449822; 31413614; 31341794; 31018749.

30548403; 30465895; 30271984; 29762637; 29269309; 29264881; 28219140; 28178319; 28071704; 27787562; 27582165; 27554111; 27551045; 27103371; 27006400; 26747726; 26553540; 26543827.

26219415; 25981634; 25825057; 25781208; 25437049; 25301875; 25301398; 25249609; 25069700; 24931083; 24607487; 24573084; 23709088; 23454124; 23438205; 23255365; 23221071; 22707199.

22693349; 22650227; 22228713; 24764509; 22124463; 22003111; 22003103; 21575160; 21357467; 20450659; 20170650; 20115927; 19463916; 19056300; 18710793; 18579779; 17641740; 17619441.

A7 Additional source checks for the present revision

The records and retrieval counts in A1–A6 are retained from the supplied manuscript as the preceding search account; they were not independently reproduced for the present update. Additional targeted web searches of PubMed-indexed primary studies and checks against accessible PubMed, PubMed Central and publisher material were undertaken on 14 September 2026. These checks constitute a focused narrative update, not a new exhaustive retrieval or an independently screened systematic review.

Representative executed queries included site.pubmed.ncbi.nlm.nih.gov metabolic memory glucose normalization epigenetic 2025 2026; site.pubmed.ncbi.nlm.nih.gov "Persistent epigenetic signals propel"; site.pubmed.ncbi.nlm.nih.gov "metabolic memory" "DNA methylation" "2020"; and exact-title checks for the SETD7, p66Shc, tubular p21, retinal DNMT and NRF2 studies. Google Scholar access was attempted with "metabolic memory" diabetes p66Shc p21 but did not return a usable results page; a completed Google Scholar search is not claimed.

Newly cited primary records are PMID 32694834 (human methylation and statistical mediation; reference 134), PMID 16249432 (skin collagen glycation and clinical outcomes; reference 135) and PMID 32313015 (retinal MFN2/MLH1 and mitochondrial stability; reference 136). The previously cited core records checked included PMIDs 18809715, 22693349, 36030260, 27787562, 34255973, 38553774 and 38755006. Details were limited to those supported by accessible primary material; missing recovery or treatment-withdrawal intervals were not invented.
